# Ablating VHL in rod photoreceptors modulates RPE glycolysis and improves preclinical model of retinitis pigmentosa

**DOI:** 10.1172/JCI185796

**Published:** 2025-02-12

**Authors:** Salvatore Marco Caruso, Xuan Cui, Brian M. Robbings, Noah Heapes, Aykut Demikrol, Bruna Lopes Da Costa, Daniel T. Hass, Peter M.J. Quinn, Jianhai Du, James B. Hurley, Stephen H. Tsang

**Affiliations:** 1 Department of Biomedical Engineering, Columbia University, New York, New York, USA.; 2 Jonas Children’s Vision Care and Bernard & Shirlee Brown Glaucoma Laboratory, Institute of Human Nutrition, Columbia Stem Cell Initiative, New York, New York, USA.; 3 Edward S. Harkness Eye Institute, Columbia University Irving Medical Center, New York-Presbyterian Hospital, New York, New York, USA.; 4 Department of Biochemistry, The University of Washington, Seattle, Washington, USA.; 5 Department of Ophthalmology and Visual Sciences and; 6 Department of Biochemistry and Molecular Medicine, West Virginia University, Morgantown, West Virginia, USA.; 7 Departments of Ophthalmology, Pathology & Cell Biology, Vagelos College of Physicians and Surgeons, Columbia University Irving Medical Center, New York, New York, USA.

**Keywords:** Metabolism, Ophthalmology, Therapeutics, Gene therapy, Glucose metabolism, Retinopathy

## Abstract

Neuroretinal degenerations including retinitis pigmentosa (RP) comprise a heterogeneous collection of pathogenic mutations that ultimately result in blindness. Despite recent advances in precision medicine, therapies for rarer mutations are hindered by burdensome developmental costs. To this end, Von Hippel-Lindau (VHL) is an attractive therapeutic target to treat RP. By ablating VHL in rod photoreceptors and elevating hypoxia-inducible factor (HIF) levels, we demonstrate a path to therapeutically enhancing glycolysis independent of the underlying genetic variant that slows degeneration of both rod and cone photoreceptors in a preclinical model of retinitis pigmentosa. This rod-specific intervention also resulted in reciprocal, decreased glycolytic activity within the retinal pigment epithelium (RPE) cells despite no direct genetic modifications to the RPE. Suppressing glycolysis in the RPE provided notable, noncell-autonomous therapeutic benefits to the photoreceptors, indicative of metabolically sensitive crosstalk between different cellular compartments of the retina. Surprisingly, targeting HIF2A in RPE cells did not impact RPE glycolysis, potentially implicating HIF1A as a major regulator in mouse RPE and providing a rationale for future therapeutic efforts aimed at modulating RPE metabolism.

## Abstract 2

Reprogramming of cell metabolism via hypoxia-inducible factors identifies mutation-agnostic therapeutic strategy for treating retinitis pigmentosa and illuminates metabolic interdependencies of cell types in the retina.

## Graphical abstract

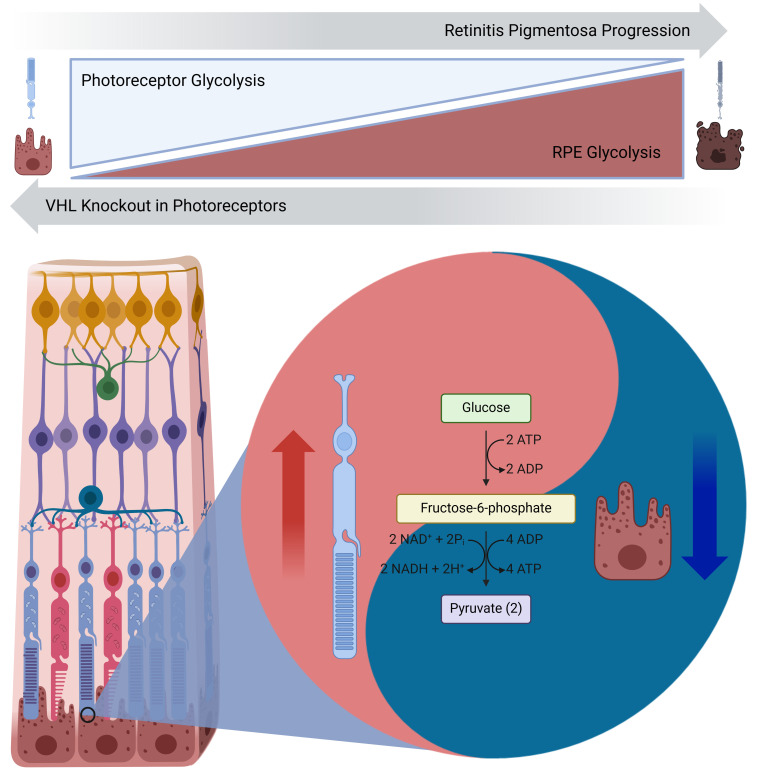

## Introduction

Ranging widely in severity and frequency, diseases causing vision loss collectively pose a global economic burden of over 100-billion USD annually (1).Retinitis Pigmentosa’s (RP’s) genetic landscape is highly heterogenous, with potentially hundreds of unique mutations for each of the more than 80 genes associated with the disorder (2). The rarity of certain mutations and the burdensome developmental costs of therapeutics have strongly inhibited clinical progress addressing these segments. A therapeutic capable of preserving retinal function independent of the underlying mutation would be both highly advantageous and desirable for treating the larger patient population. In recent years, several independent researchers have demonstrated that aging and dystrophic photoreceptors exhibit a metabolic compartmental shift from glycolysis to oxidative phosphorylation (OXPHOS) across multiple genetic backgrounds while their retinal pigment epithelium (RPE) counterparts shift simultaneously to increase reliance on glycolysis and decrease reliance on OXPHOS (3–6).

In a young healthy eye, rods take up glucose from RPE and convert it into lactate in the presence of oxygen, a phenomenon also known as aerobic glycolysis or the Warburg effect (7–9). A paracrine factor from the rods is then used as a substrate for OXPHOS in RPE and a suppressor of RPE-specific glucose consumption (5, 10). While rod and cone photoreceptors rely primarily on glucose for energy, the RPE can oxidize a broad range of substrates including lactate, glucose, succinate, proline, or ketones (10, 11). Altogether, a potential metabolic coupling dynamic emerges where the RPE acts as a niche compartment that fuels the glycolytic rods and potentially vice versa. When this metabolic balance becomes asynchronous, the retinal ecosystem begins to fail—a decrease in lactate concentration due to rod death may prompt an increase in RPE glucose consumption, possibly depriving photoreceptors of their preferred metabolic fuel (10). This potential decompartmentalization toward aerobic glycolysis in RPE and OXPHOS in rods underlies cone starvation, death, and ultimately blindness across a variety of retinitis pigmentosa genetic backgrounds (12–14).

While there are many potential avenues for reprogramming glycolysis, targeting hypoxia-inducible factors (HIFs) remains particularly attractive due to their broad roles in regulating cell metabolism. HIFs act as regulatory nodes of glycolysis in response to hypoxia, resulting in a robust protective remodeling that generally supports energetic demands by moving away from oxygen-dependent OXPHOS. Particularly, HIF1A directs glucose away from OXPHOS toward anaerobic glycolysis, which may free up carbon from glucose for anabolic activity (15, 16). Under normoxic conditions, HIFs are rapidly degraded by prolyl hydroxylation (PHD) and ubiquitination stimulated by Von Hippel-Lindau (VHL). VHL is the recognition component of an E3 ubiquitin ligase complex that targets HIF1A/HIF2A for proteolysis (16). Photoreceptors in the retina can thrive in a low-oxygen environment (6). However, in retinitis pigmentosa, hyperoxia (driven by diminished O_2_ consumption) may stimulate further retinal degeneration (17–19).

Like other conserved cellular mechanisms of degeneration, reprogramming of cell metabolism can slow the progression of retinitis pigmentosa (20–26). Here, we ablated VHL specifically in the rods to evaluate the effects of stabilizing HIFs, which are downregulated in the aging and diseased retina (27, 28). We hypothesize that enhancing glycolysis via stabilization of HIF levels can delay degeneration by rejuvenating metabolism in diseased photoreceptors. Following ablation of VHL, we observed a stabilization of HIFs and robust functional and histological rescues. These findings were supported by increased glycolytic intermediates in the retina. Unexpectedly, we also observed a robust remodeling of RPE metabolism following rod-specific ablation of VHL, supporting a metabolic crosstalk between cell compartments. To assess if suppressing glycolysis in the RPE would also provide therapeutic benefit, we explored the impact of HIF2A ablation on degeneration. Our findings suggest that HIF-mediated rescues may involve pathways in addition to cell metabolism and identify HIF1A as a future therapeutic targeting for modulating metabolism in the RPE.

## Results

To probe the effects of a rod-specific VHL ablation, we crossed *Vhl^loxP/loxP^* mice (JAX 004081; *Vhl^tm1Jae^/J* mice) with a *Pde6g^Cre–ERT2/+^* line (29, 30). The Cre recombinase allele was kept heterozygous to avoid interfering with the endogenous gene’s function. This cross yielded *Vhl^loxP/loxP^*; *Pde6g^Cre–ERT2/+^* mice, which were then crossed with our *Pde6b^H620Q/H620Q^* RP mice to generate our experimental *(Vhl^–/–^; Pde6b^H620Q/H620Q^; Pde6g^Cre–ERT2/+^*) and control mice *(Vhl^loxP/loxP^*; *Pde6b^H620Q/H620Q^; Pde6g^Cre–ERT2/+^*). In our experimental line, tamoxifen treatment subsequently activated the Cre recombinase, which then excised a region of the *VHL* promoter and exon 1, silencing gene expression (29). The resulting model demonstrated a robust retinal degeneration, characterized by histological thinning and functional electroretinogram (ERG) deficits, which is consistent with previous studies of the *Pde6b^H620Q/H620Q^* variant (21). Experimental mice were injected with tamoxifen on 3 separate days ranging from 7 to 10 days postnatal (P7–P10). Successful tamoxifen-dependent genomic excision in rod photoreceptors was demonstrated by the presence of a truncated PCR product (Supplemental Figure 1A; supplemental material available online with this article; https://doi.org/10.1172/JCI185796DS1), a decrease in VHL protein levels, an increase in HIF1A/HIF2A proteins, and an increase in downstream targets (Supplemental Figure 1B). Analysis was performed at 3 weeks of age to mitigate obfuscation and variability associated with degeneration.

**Figure 1:**
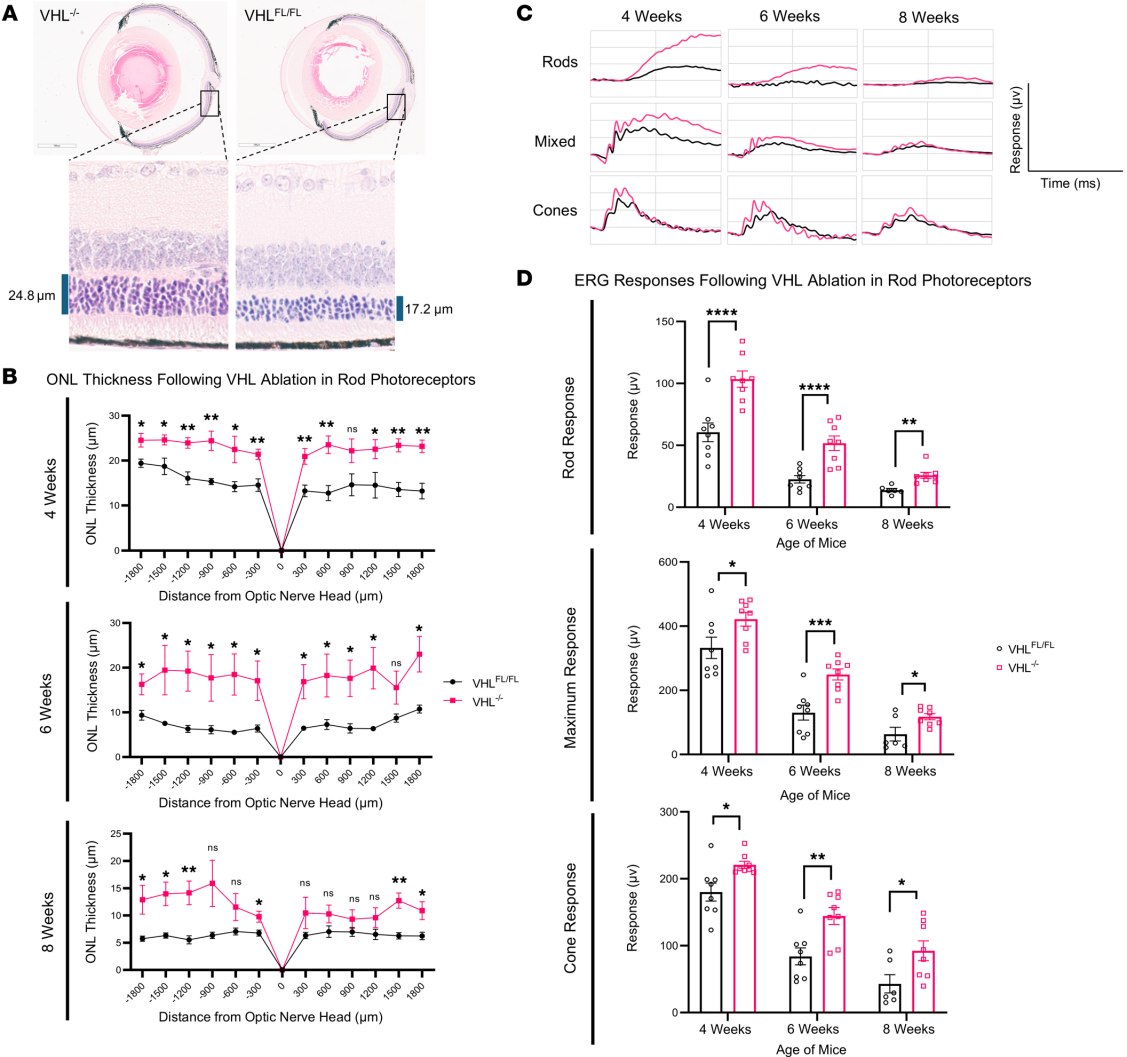
Rod-specific VHL ablation improves both rod and cone function in the *Pde6b*^H620Q/H620Q^ mutant background. Cre-inducible treated (*Vhl^–/–^; Pde6b^H620Q/H620Q^*; *Pde6g^Cre–ERT2/+^*) and untreated (*Vhl^loxP/loxP^; Pde6b^H620Q/H620Q^*; *Pde6g^Cre–ERT2/+^*) mice were subjected to functional and histological analyses that demonstrated meaningful rescue. (**A**) Representative schematic of histological cross sections from treated and untreated eyes is shown. (**B**) Quantification of retinal thickness as a distance from optic nerve as measured from histological sections (*n* ≥ 5). A 2-tailed *t* test was performed to determine significance. (**C**) Representative traces of treated (pink) and control (black) mice at each timepoint for each phase are provided. ERG analysis consisted of 3 phases: a dark-adapted phase in which the rod-specific responses were studied, a maximum response where full retinal signaling occurs, and a cone-specific response in which the rods are photobleached. (**D**) ERG responses of 4-, 6-, and 8-week-old mice (*n* ≥ 6). An average across the eyes was taken for each mouse and treated as *n* = 1. A 2-tailed *t* test was performed to determine significance. All Error bars shown represent mean ± SEM. *P < 0.05; **P < 0.01; ***P < 0.001; ****P < 0.0001.

**Figure 2:**
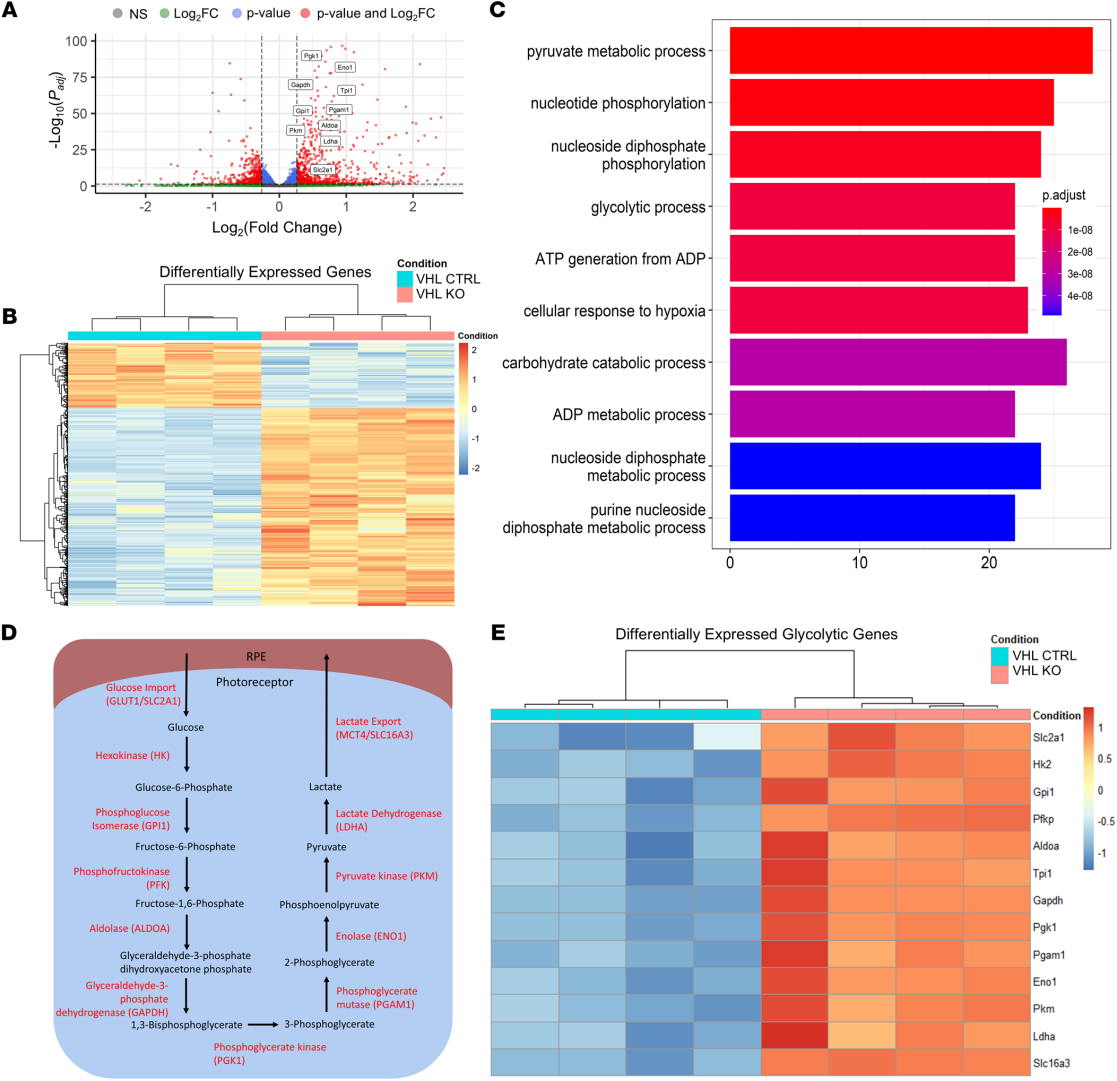
VHL ablation in photoreceptors results in glycolytic reprogramming and robust transcriptional remodeling. VHL ablation resulted in a robust transcriptional remodeling of photoreceptor behavior, altering several biological processes, including the regulation of metabolic processes, cellular responses to hypoxia, and ATP generation. (**A**) Volcano plot of DESeq2 results comparing VHL ablated (*Vhl^–/–^; Pde6b^H620Q/H620Q^*; *Pde6g^Cre–ERT2/+^*, *n* = 4) and unablated (*Vhl^loxP/loxP^; Pde6b^H620Q/H620Q^*; *Pde6g^Cre–ERT2/+^*, *n* = 4) neuroretina identified a variety of DEGs, including glycolytic intermediates such as *Glut1*, *Hk1*, *Pkm*, and *Ldha*. (**B**) Heatmap of significant DEGs identified a wide range of targets both up and down regulated following intervention. (**C**) Gene ontology analysis (GO) performed on DEGs identified a wide range of biological processes implicated in the knockout including glycolysis, hypoxia responses, and ATP synthesis (**D**) Schematic of glycolysis pathway and enzymes associated with each reaction. (**E**) DEGs demonstrating upregulation of glycolysis and TCA cycle as identified by RNA-seq analysis at 3 weeks of age comparing VHL ablated and unablated mice. Statistics were performed in DESeq2 using Wald tests and multiple comparisons were accounted for using the Benjamini-Hochberg method to control the FDR.

Tamoxifen-injected mice were evaluated at 4, 6, and 8 weeks of age for histological and functional metrics. These endpoints were significantly improved over untreated counterparts up to nearly 2 months (Figure 1). Rod-specific ablation of VHL improved photoreceptor preservation, as measured by outer nuclear layer (ONL) thickness as a function of distance from the optic nerve (*n* ≥ 5, Figure 1, A and B). VHL ablation also resulted in significant ERG improvements across all 3 phases (rod-specific, maximum, and cone-specific responses) at all timepoints evaluated (*n* ≥ 6, Figure 1, C and D). To define the mechanism of rescue involved in VHL’s ablation, we performed RNA-seq analysis on neural retina at 3 weeks of age, prior to the onset of degeneration (*n* = 4). DESeq2 analysis identified a variety of differentially expressed genes (DEGs), including downregulation of *Vhl* and an upregulation of glycolytic proteins/enzymes, including glucose transporter 1 (*Glut1*), hexokinase 1/2 (*Hk1*/*Hk2*), glyceraldehyde 3-phosphate dehydrogenase (*Gapdh*), enolase I (*Eno1*), pyruvate kinase (*Pkm*), and lactate dehydrogenase A (*Ldha*) (Figure 2, A and B). Further analysis showed upregulation of *Pdk1*, a master regulator of pyruvate fate that inhibits uptake into the mitochondria for OXPHOS and thus promotes aerobic glycolysis. Similarly, *Pdk3* was also upregulated, which inhibits the conversion of pyruvate into acetyl-CoA, an essential early step of OXPHOS. The major lactate exporter *Mct4* was also upregulated. Gene ontology analysis was performed to assess the effect of these changes and identified a variety of biological processes in line with our hypothesis, including metabolic processes, biogenesis, and cellular responses to hypoxia (Figure 2C). For convenience, a schematic of the glycolysis pathway and enzymes associated with each reaction has been included in Figure 2D. Upstream glycolytic enzymes regulating the metabolism of glucose into lactate were widely upregulated in the treated group (Figure 2E). VHL and glycolytic DEGs identified by RNA-seq were confirmed via qPCR. *Vhl* mRNA similarly decreased while *Glut1* and glycolytic enzymes such as *Hk1* and *Ldha* were also transcriptionally upregulated (Supplemental Figure 1C). Supporting analyses of bulk RNA-seq are shown in Supplemental Figure 2.

**Figure 3:**
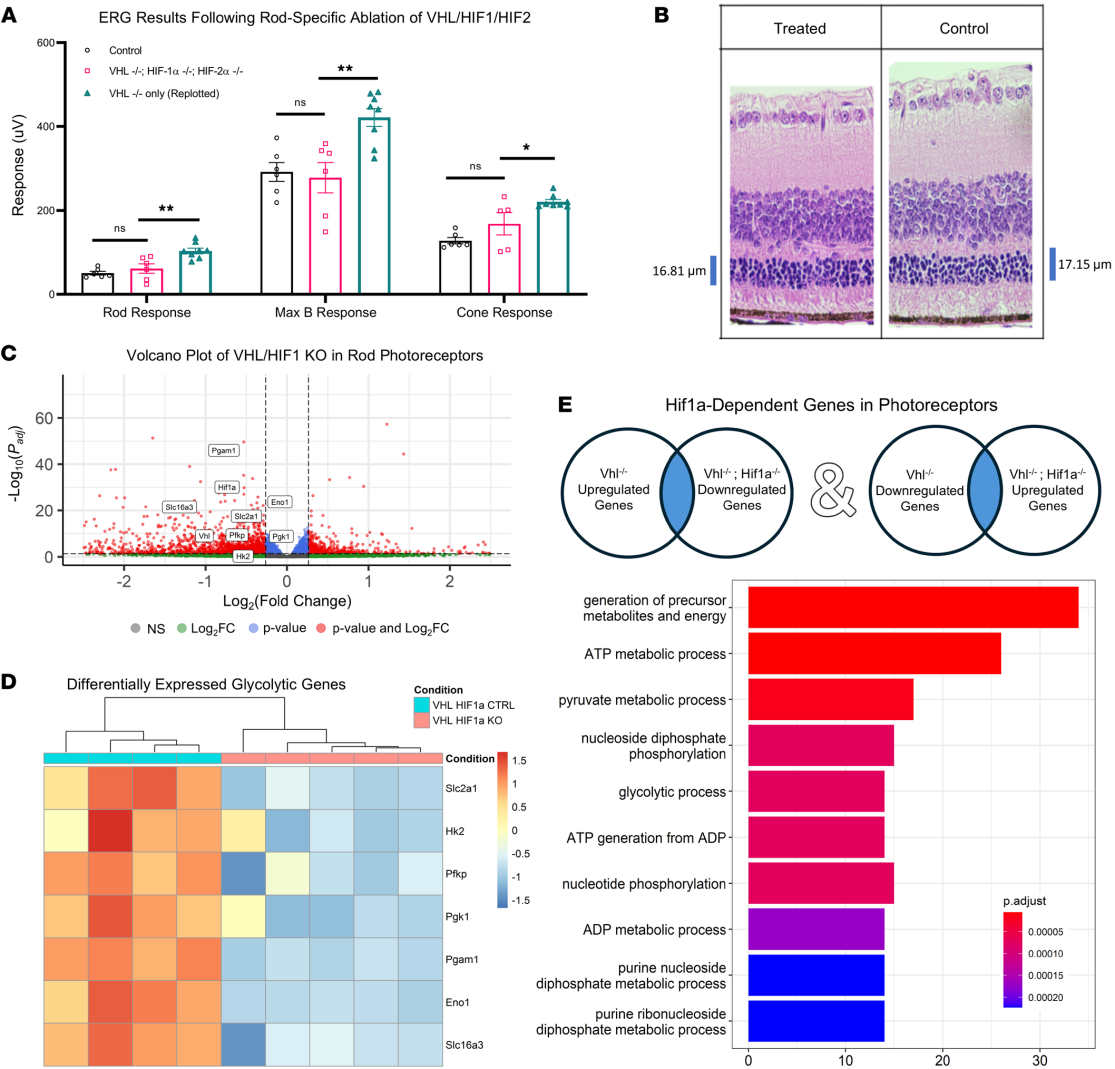
Rod-Expression of HIFs are essential for VHL-mediated rescue and transcriptional enhancement of glycolysis. RNA-seq identified increased glucose transporters and key enzymatic intermediates responsible for regulating glycolysis. The observed therapeutic effects were likely driven by HIFs and were negated following triple KO of VHL/HIF1A/HIF2A in rod cells, with metabolic changes highly dependent on HIF1A expression. (**A**) ERG analysis at 4 weeks of age comparing VHL/HIF1A/HIF2A triple knockout (*Vhl^–/–^; Hif1a^–/–^; Hif2a^–/–^; Pde6b^H620Q/H620Q^*; *Pde6g^Cre–ERT2/+^*, *n* = 6) and control (*Vhl^loxP/loxP^; Hif1a^loxP/loxP^; Hif2a^loxP/loxP^; Pde6b^H620Q/H620Q^*; *Pde6g^Cre–ERT2/+^*, *n* = 6) mice demonstrated a complete loss of therapeutic effects associated with VHL ablation. Statistical analysis was performed using ANOVA to determine if a difference existed between groups followed by post hoc Tukey’s Honest Significant Difference (HSD) test. (**B**) Histological cross sections comparing triple-KO and control mice demonstrated similar findings with no difference in ONL thickness between treated and untreated mice. (**C**) Volcano plot of bulk RNA-seq results comparing VHL/HIF1A-ablated (*Vhl^–/–^; Hif1a^–/–^; Pde6b^H620Q/H620Q^*; *Pde6g^Cre–ERT2/+^*) mice and control mice (*Vhl^loxP/loxP^; Hif1a^loxP/loxP^; Pde6b^H620Q/H620Q^*; *Pde6g^Cre–ERT2/+^*). (**D**) Highlighted findings of bulk RNA-seq demonstrating downregulation of several glycolytic enzymes following VHL/HIF1A ablation, including *Glut1* and *Pfk*. Statistics were performed in DESeq2 using Wald tests and multiple comparisons were accounted for using the Benjamini-Hochberg method to control the FDR. (**E**) GO analysis of HIF1A-dependent genes (i.e., those inversely regulated in VHL and VHL/HIF1A KOs) demonstrated a strong regulation of glycolysis by HIF1A. All Error bars shown represent mean ± SEM. * *P* < 0.05; ** *P* < 0.01.

To determine whether the observed rescue was through the proposed HIF axis, we crossed experimental mice into a previously described *Hif1a^loxP/loxP^*, *Hif2a^loxP/loxP^* background, creating the new model: (*Vhl^loxP/loxP^*; *Hif1a^loxP/loxP^*; *Hif2a^loxP/loxP^*; *Pde6b^H620Q/H620Q^; Pde6g^Cre–ERT2/+^*). Tamoxifen injections at identical time points as previous work led to excision of VHL and the essential α subunits of HIF-1 and HIF-2 (31). No rescue effect was observed following treatment, as measured by ERG and histology, indicating that HIFs are essential for the rescue effects observed following VHL ablation (Figure 3, A and B). In parallel, we also explored the importance of HIF1A within the context of metabolic reprogramming, performing bulk RNA-seq on 3-week-old degenerative mice with VHL and HIF1A-ablated (*Vhl^–/–^*, *Hif1a^–/–^*, *Pde6b^H620Q/H620Q^; Pde6g^Cre–ERT2/+^*) and control mice (*Vhl^loxP/loxP^*, *Hif1a^loxP/loxP^*, *Pde6b^^H620Q/H620Q^^; Pde6g^Cre–ERT2/+^*). Combinatory VHL/HIF1A ablation in the photoreceptors not only offset the increased glycolysis observed in the VHL-KO mice, but also meaningfully suppressed glycolytic intermediates (Figure 3, C and D), emphasizing the regulatory role of HIF1A in photoreceptor metabolism. *Pdk1* and *Mct4* were also downregulated in response to the knockout. GO analysis of HIF1A-dependent genes (i.e., genes upregulated in VHL KO and downregulated in VHL/HIF1A KO, or vice versa) identified an enrichment of various metabolic processes, including glycolysis (Figure 3E). Supporting analyses are shown in Supplemental Figure 3.

While ablating VHL provides therapeutic effects in a mouse model of retinitis pigmentosa, upregulation of HIFs may also promote neovascularization in response to elevated vascular endothelial growth factors (VEGFs) induced by HIFs. We performed angiograms on rod-specific, VHL ablated (*Vhl^–/–^; Pde6b^H620Q/H620Q^; Pde6g^Cre–ERT2/+^*), and control (*Vhl^loxP/loxP^*; *Pde6b^^H620Q/H620Q^^; Pde6g^Cre–ERT2/+^*) mice at 3 weeks of age. Angiography suggested normal vasculature compared with age-matched controls (Supplemental Figure 4A). These results confirm the findings of previous studies that rigorously assessed the impact of VHL ablation on vasculature in both rods specifically and the greater neuroretina (32, 33). Lastly, a cohort of mice was taken out to 18 months of age and analyzed for any histological abnormalities, including hemangioblastomas. No adverse events were detected (Supplemental Figure 4B).

**Figure 4:**
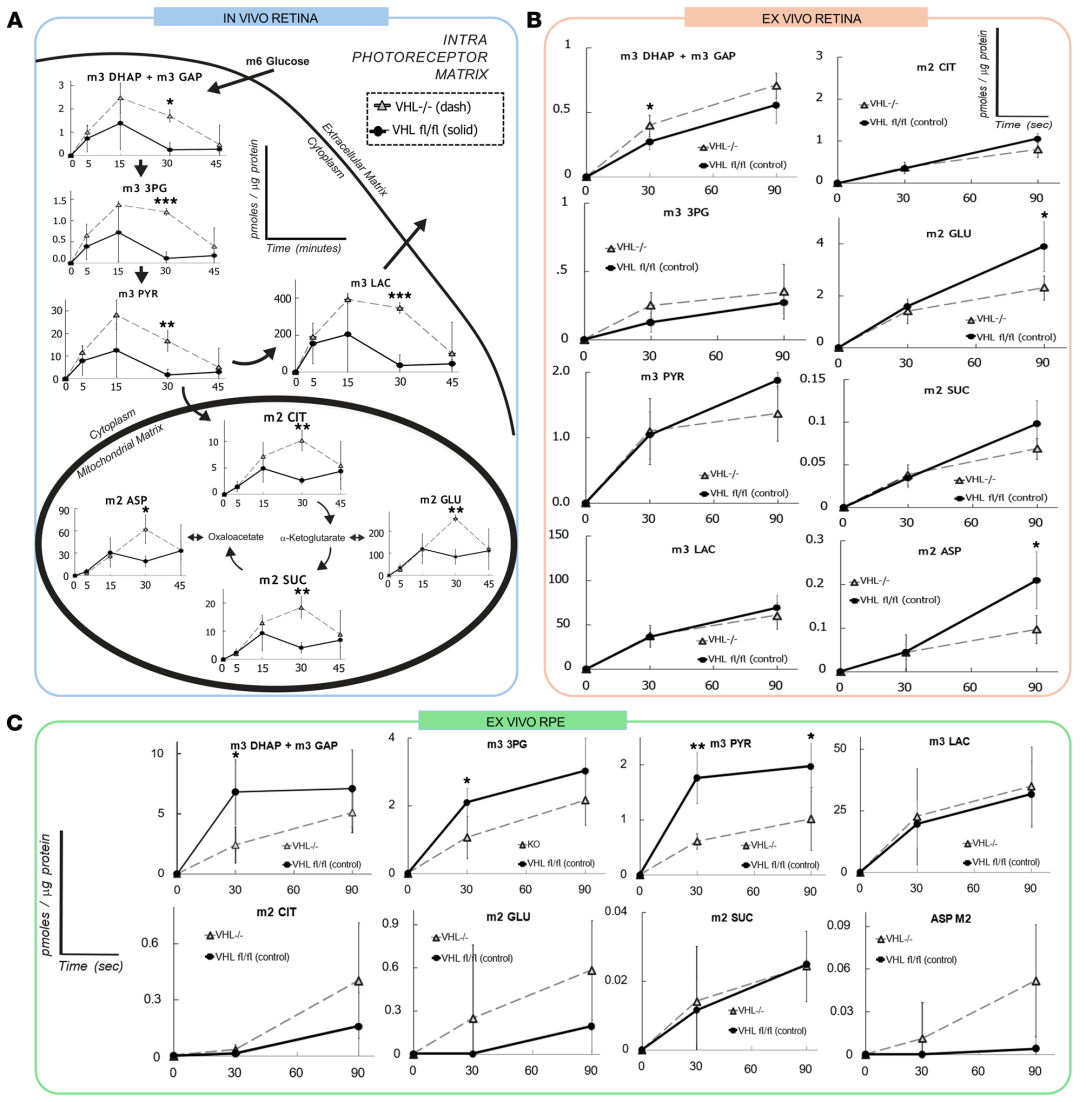
VHL ablation in rods causes increased glycolysis in photoreceptors, accompanied by a reciprocal reduction in glycolysis in nongenetically modified RPE cells. VHL ablation in rods resulted in increased glycolysis in the photoreceptors, in addition to a suppressed glycolysis in the RPE indicative of a potential crosstalk mechanism. (**A**) In vivo targeted analysis of glycolysis and oxidative phosphorylation in the photoreceptors of treated (*Vhl^–/–^; Pde6b^H620Q/H620Q^*; *Pde6g^Cre–ERT2/+^*) and untreated (*Vhl^loxP/loxP^; Pde6b^H620Q/H620Q^*; *Pde6g^Cre–ERT2/+^*) mice at 3 weeks of age. An upregulation of glycolytic intermediates was observed following VHL ablation, in addition to increases in oxidative phosphorylation intermediates (*n* = 3). (**B**) Ex vivo targeted analysis of the retina comparing treated and untreated mice. Instead of receiving glucose supply from the RPE in vivo, photoreceptors were allowed to readily uptake glucose from the surrounding media (*n* = 4). (**C**) Ex vivo targeted analysis of RPE/Choroid tissues following rod-specific VHL ablation. Despite being unedited, RPE cells experienced decreased glycolysis and increased mitochondrial flux (*n* ≥ 4). A 2-tailed *t* test was performed to determine significance in each of the graphs. All error bars shown represent mean ± SEM. * *P* < 0.05; ** *P* < 0.01; *** *P* < 0.001.

Stabilization of HIFs by blocking VHL expression increased levels of glycolytic proteins, suggesting they may alter metabolic flux in the retina. To test this idea, we injected mice with U-^^13^^C-glucose (500 mg/kg, intraperitoneal) at P21. We euthanized mice 5, 15, 30, or 45 minutes following the injection, isolated retinas, and snap froze them in liquid nitrogen. We extracted and derivatized metabolites, then determined metabolite levels and the distribution of ^^13^^C from U-^^13^^C-glucose using gas chromatography-mass spectrometry (GC-MS). We observed a significant increase in the abundance of m+3 labeled 3-phosphoglycerate (3PG), pyruvate, and lactate in the VHL-ablated group (Figure 4A), suggesting enhanced glycolytic flux. Accumulation of ^^13^^C on TCA cycle intermediates was also increased, though not as strongly as the increase in retention of ^^13^^C in glycolysis. To determine if the increase in flux from glucose was intrinsic to the retina, we incubated dissected retina tissue in 5 mM U-^^13^^C glucose for 30 or 90 seconds. While glucose uptake to photoreceptors is regulated by the RPE in vivo, U-^^13^^C glucose from media is instead imported directly into the dissected retinal tissue. Under these conditions, VHL ablation had a more limited but present effect on metabolic flux (Figure 4B). Flux into DHAP, GAP, and 3PG increased and flux through intermediates synthesized in mitochondria decreased.

Remarkably, VHL deficiency in rods influenced the metabolic features of RPE/choroid even after the RPE/choroid tissue had been removed from the eye. Figure 4C shows flux from ^^13^^C glucose in RPE choroid tissue that had been isolated from eyes of mice with VHL-ablated rods. Flux through glycolysis appeared slower than normal, while flux through mitochondrial intermediates was faster. Meanwhile, Supplemental Figure 5 provides statistical confirmation that RPE/choroid from eyes with VHL-deficient rods support greater-than-normal TCA cycle flux. These unexpected findings suggest a crosstalk between the retina and RPE that can alter the supply of nutrients delivered to the retina. The rescue of photoreceptors by rod-specific VHL ablation may be due partially to this photoreceptor-RPE crosstalk positively contributing to a strategy for treating retinitis pigmentosa.

Given the therapeutic benefit of VHL ablation in the rods, we hypothesized that HIF ablation in the RPE could increase the flow of nutrients to photoreceptors, providing a similar mechanism of rescue. While we observed HIF1A to play a major role in the regulation of glycolysis in photoreceptors, it is unknown if this relationship will be conserved in the RPE of mice. Several groups have previously identified HIF2A as a major driver of degeneration in the RPE (6, 34–36). Furthermore, analysis of human stem cell–derived RPE (iRPE) performed by our team treated under hypoxic conditions demonstrated a clear increase in glycolytic intermediates positively correlated with elevated HIF2A expression, not HIF1A (37). Consequently, we elected to study the potential therapeutic impact of HIF2A ablation in the RPE. To explore this concept, we generated mice that retained the *Pde6*b*^H620Q/H620Q^* genotype responsible for pathology and introduced a floxed *Hif2a* gene by crossing with a previously published mouse model from Jackson Laboratory (JAX 008407) (38). To allow for RPE-specific ablation, we crossed this line with an inducible RPE65-driven CreERT2 allele, achieving the final genotype: *Hif2a^loxP/loxP^*; *Pde6b^H620Q/H620Q^*; *Rpe65^P2A–Cre–ERT2/+^* (39).

**Figure 5:**
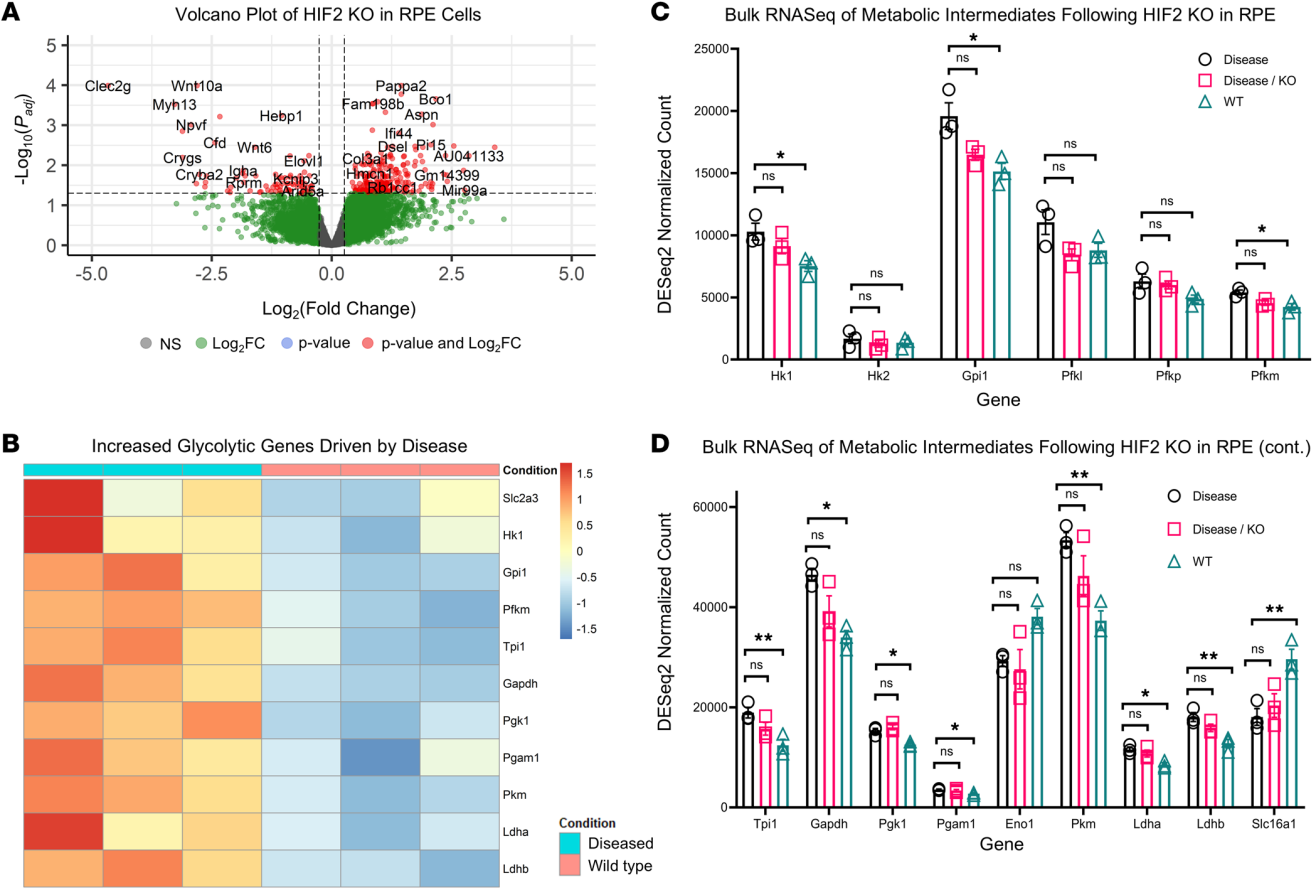
RPE-specific ablation of HIF2A does not alter metabolic transcriptome. RPE-specific ablation of HIF2A failed to reverse the increase in glycolytic enzyme transcripts observed in response to disease progression. (**A**) Volcano plot of bulk RNA-seq comparing RPE-specific HIF2A-KO mice (*Hif2a*^–/–^; *Pde6b^H620Q/H620Q^*; *Rpe65*^P2A–Cre–ERT2/+^) to control mice (*Hif2a^loxP/loxP^; Pde6b^H620Q/H620Q^*; *Rpe65^P2A–Cre–ERT2/+^*). (**B**) DESeq results of glycolytic intermediates comparing WT control mice and degenerative mice (*Hif2a^loxP/loxP^; Pde6b^H620Q/H620Q^*; *Rpe65^P2A–Cre–ERT2/+^*) confirms previously published findings regarding elevated glycolysis in the RPE associated with retinitis pigmentosa disease progress. (**C**) Comparison of normalized counts between WT (green), diseased (black), and diseased mice treated via HIF2A ablation in the RPE (pink). No change was observed in upstream or (**D**) downstream glycolytic transcripts when compared between diseased control (black) and treated (pink) (*n* = 3). Statistics were performed in DESeq2 using Wald tests and multiple comparisons were accounted for using the Benjamini-Hochberg method to control the FDR. Error bars represent mean ± SEM. * *P* < 0.05; ** *P* < 0.01.

**Figure 6:**
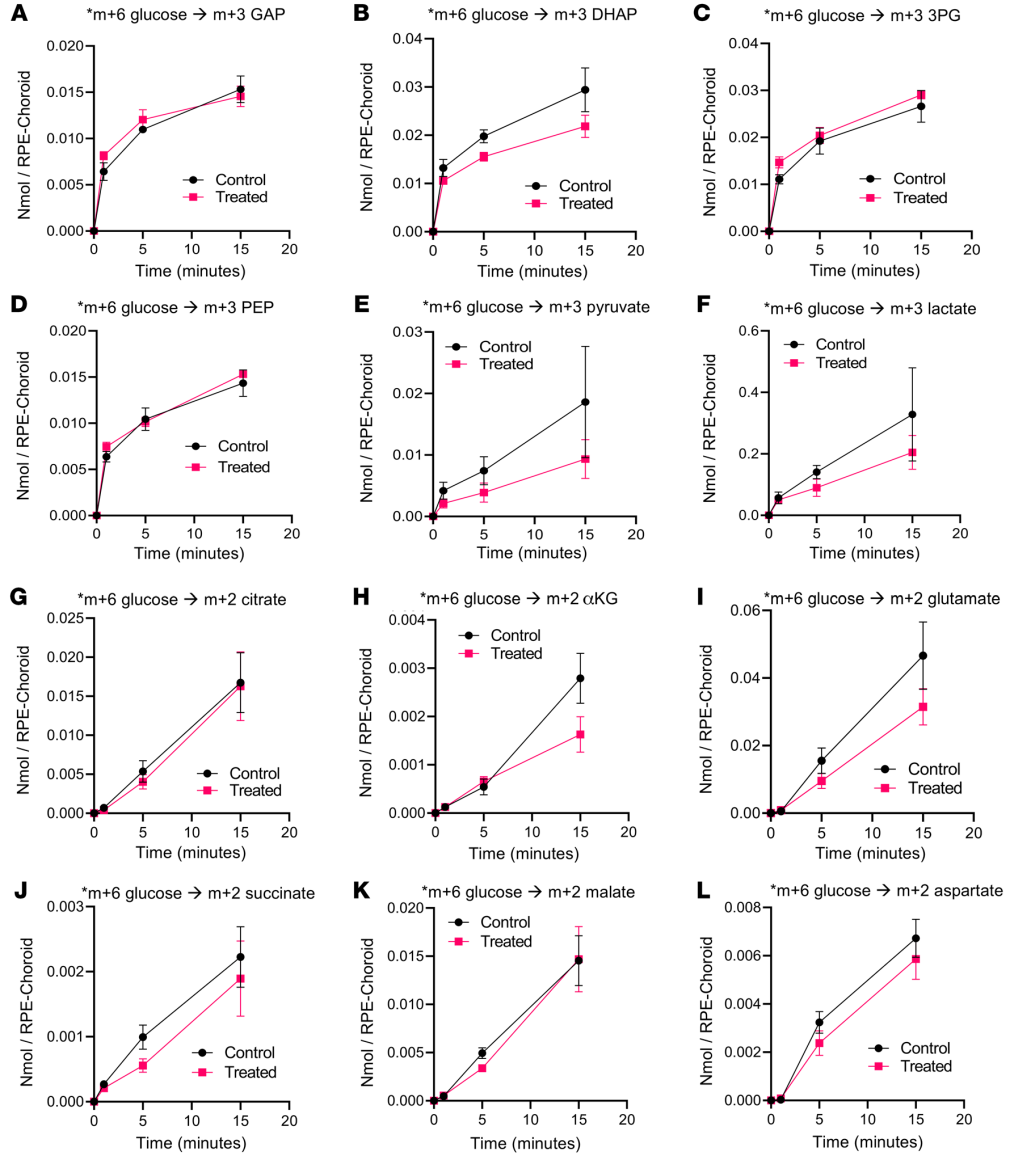
HIF2A maintains canonical independence from glycolysis in the RPE of degenerative mice. RPE-specific ablation of HIF2A did not alter RPE-choroid glucose metabolism ex vivo. RPE-choroid tissue was dissected from 21-day-old experimental (*Hif2a*^–/–^; *Pde6b^H620Q/H620Q^*; *Rpe65*^P2A–Cre–ERT2/+^) and control (*Hif2a^loxP/loxP^; Pde6b^H620Q/H620Q^*; *Rpe65^P2A–Cre–ERT2/+^*) mice, then incubated in 5mM U-^13^C glucose for 1, 5, or 15 minutes. Following the incubation, retina tissue was flash-frozen in liquid nitrogen. Metabolites were extracted, derivatized, and run on a GC-MS system. Accumulation of ^13^C to form (**A**) m+3 glyceraldehyde3-phosphate (GAP), (**B**) m+3 dihydroxyacetone phosphate (DHAP), (**C**) m+3 3-phosphoglycerate (3-PG), (**D**) m+3 phosphoenolpyruvate (PEP), (**E**) m+3 pyruvate, (**F**) m+3 lactate, (**G**) m+2 citrate, (**H**) m+2 α-ketoglutarate (αKG), (**I**) m+2 glutamate, (**J**) m+2 succinate, (**K**) m+2 malate, and (**L**) m+2 aspartate was unchanged (*n* = 3–4). Error bars represent mean ± SEM.

Downregulation of HIF2A following injection was verified at the genomic, transcriptomic, and proteomic levels, in addition to well-known downstream targets (Supplemental Figure 6). As HIF isoforms may have redundant roles, we also performed immunoblots for HIF1A and observed comparable proteomic levels following HIF2A KO (Supplemental Figure 6). 3-week-old HIF2A ablated and unablated RPE were analyzed by bulk RNA-seq in addition to a WT, nondegenerative, age-matched control (Figure 5A). As expected, bulk RNA-seq identified a substantial increase in glycolysis associated with the degeneration compared with the healthy WT samples (Figure 5B) and confirmed modulation of both HIF2A and known HIF2A targets in HIF2A-ablated mice. However, bulk RNA-seq did not identify any changes in glycolytic enzymes between HIF2A-ablated and -unablated degenerative samples (Figure 5, C and D, supporting analyses in Supplemental Figure 7). To confirm if RPE-specific HIF2A ablation failed to alter RPE metabolism, we incubated ex vivo RPE-choroid tissue in U-^^13^^C-glucose for 1, 5, and 15 minutes. The flux of ^^13^^C from glucose to downstream glycolytic and TCA cycle intermediates was unaltered (Figure 6, A–L), supporting the canonical independence between HIF2A and glycolysis in mouse RPE. Flux to aspartate may have been impaired by HIF2A deletion, so we probed aspartate synthesis from the late TCA cycle by providing RPE choroid with U-^^13^^C-pyruvate or U-^^13^^C-succinate for 90 seconds. The flux of ^^13^^C- succinate to aspartate appeared increased, consistent with a trend of enhanced mitochondrial activity in the HIF2A-KO RPE-choroid (Supplemental Figure 8).

While HIF2A ablation failed to achieve metabolic reprogramming, we observed no additional degeneration associated with the intervention (Supplemental Figure 9), as determined by ERG and histology. To determine if the composition of rods and photoreceptors within the ONL had changed despite minimal changes in overall ONL thickness, we counted the cones marked with antipeanut agglutinin on flat mounts from HIF2A KO at approximately 10 weeks old (*n* = 6 treated, *n* = 4 untreated). While the average number of cones in the treated group was higher than in the untreated group across the entire retina, this difference did not achieve statistical significance. As such, the ablation of HIF2A in the RPE, while unable to achieve metabolic reprogramming on its own, did not exacerbate retinal degeneration and may potentially offer some mild benefits.

## Discussion

There are limited therapeutic options currently for patients with retinal degenerations, with only a single FDA-approved gene therapy to treat a narrow subset of patients with mutations in the *RPE65* gene (Luxturna) (40). Given the heterogeneity of retinitis pigmentosa and its rarity, precision medicine approaches aimed at fixing each mutation, such as gene editing, may be prohibitively cost-inefficient and time consuming. Meanwhile, roughly 1-in-3 patients with inherited retinal diseases (including RP) do not have an identifiable genetic cause (13). To develop a commercially viable therapeutic strategy, we have investigated an approach targeting a universally conserved pathway of photoreceptor degeneration — glycolysis. In targeting this mechanism independent of the underlying mutation, our goal is to preserve cone function equivalent to more than 15 years at a substantially reduced cost when compared with multiple gene- or mutation-specific treatments (26, 41).

Naturally, rod photoreceptors receive glucose via the RPE and metabolize it into lactate via aerobic glycolysis even in the presence of oxygen (Warburg effect). The lactate produced by the retina may be used as a substrate for oxidative phosphorylation in the RPE and a suppressor of RPE-specific glycolysis (5). However, in retinitis pigmentosa and potentially in other rod dystrophies, the metabolic states of both the RPE and photoreceptors deviate from basic biological roles, starving the photoreceptors of essential nutrients like glucose (3, 42–44). HIFs broadly regulate a wide range of biological processes in response to hypoxia. Manipulating HIF expression may be protective against disease progression and effective strategy for generalizable therapeutics (45). VHL regulates a large network of downstream metabolic genes, making it an exceptional candidate for CRISPR therapeutics or pharmacological inhibitors (46). While PHD also remains an interesting target for overexpressing HIFs and has been successfully targeted for metabolic reprogramming within the retina, it is unknown which target of the 2 will prove more druggable and capable of eliciting a greater therapeutic effect (47). Furthermore, PHD and VHL have unique interactomes that may positively hinder or promote therapeutic responses, thus requiring their independent explorations. Here, we described the therapeutic benefit of ablating VHL and upregulating HIFs specifically in the photoreceptors, both delaying cell death and preserving vision (Figure 1). These protective effects may be explained in part by successful reprogramming of photoreceptor metabolism in a HIF-dependent manner while, surprisingly, also tuning RPE metabolism.

To determine if the rescue effect observed was associated with the proposed metabolic reprogramming, we performed a targeted analysis of glycolytic and oxidative phosphorylation intermediates. Transcriptomic analysis identified a wide range of upregulated glycolytic enzymes at 3 weeks of age following intervention (Figure 2E). Additional experiments identified HIFs as drivers of the observed rescue, with HIF1A strongly implicated in the regulation and mediation of glycolytic reprogramming. As few genes were identified to be both positively upregulated or downregulated in VHL and VHL/HIF1A knockouts (i.e., significantly altered independently of HIF1A expression), the contributions of HIF2A may be minor but must be studied more extensively before any definitive conclusions can be made. Following an injection of U-^13^C-labeled glucose, in vivo metabolomics similarly identified a stark increase in ^13^C-labeled glycolytic intermediates in VHL-ablated retinas (Figure 4A).

Unexpectedly, when photoreceptors were allowed to bypass the RPE and uptake glucose directly from media in ex vivo analyses, the metabolic impact of VHL ablation was attenuated, suggesting that rod-specific VHL ablation affects retinal metabolism also through nonautonomous metabolic changes to the RPE. Such reciprocal metabolic coupling is confirmed by the flux analysis of the ex vivo RPE, where we observed a meaningful decrease in ^13^C-labeling of glycolytic intermediates and an increase in TCA flux following VHL ablation. Rods release a wide range of paracrine factors that perform essential biological functions, including cone protection (RdCVF), ganglion cell repair (VEGF), and angiogenesis inhibition (PEDF) (48–50). These results from our targeted metabolic analysis suggest a mechanism of crosstalk between the photoreceptors and RPE, where photoreceptors communicate and alter RPE metabolism to better support retinal energy production. It is important to note that metabolomic analyses were performed on bulk retinae. While the neuroretina is predominately comprised of photoreceptors, a limitation of this work is that other cell populations are also included in analysis. Consequently, future single-cell metabolomics may confirm our findings and provide a deeper understanding of the metabolism of various retinal cell types.

Given the potential promise of VHL as a therapeutic target as well as the metabolic interdependencies of photoreceptors and RPE cells, we were determined to see if this crosstalk and rescue was reciprocal — namely, if modulating glycolysis in the RPE would produce a similar degree of rescue while also increasing glycolysis in the photoreceptors. As increasing HIFs to increase glycolysis in the photoreceptors proved therapeutic, we hypothesized that HIF suppression in the RPE should accomplish the inverse, downregulating glycolysis and allowing more glucose to reach the starving photoreceptors. Given previous work studying HIFs in the RPE, we elected to study HIF2A for its potential link to cell metabolism in this specific biological context.

In line with previously published literature, we observed an increase in RPE glycolytic enzymes driven by the disease relative to healthy, nondiseased controls. However, metabolism was surprisingly unaffected by loss of HIF2A in the context of retinitis pigmentosa, as shown by bulk RNA-seq (Figure 5) and metabolic tracings (Figure 6). These findings support the canonical independence of HIF2A and glycolysis that has been previously reported in a variety of biological contexts. While unable to achieve the goal of metabolic reprogramming, the data provides rationale for future efforts aimed at modulating HIF1A, which has historically proven challenging to target in an isoform-specific manner. Pharmacological efforts to achieve specific ablation of HIF1A generally rely on upstream interventions that increase the risk of unintended alterations (51). HIF-specific, but isoform-nonspecific, inhibitors are readily available but have been limited in their applications due to their inability to discriminate isoforms. These data demonstrate that HIF2A can be inactivated in the RPE within the context of retinitis pigmentosa without detriment, supporting the rationale for future delivery of isoform-nonspecific HIF inhibitors to the RPEs.

The presence of glycolytic imbalance in a broad spectrum of neurodegenerative diseases, including age-related macular degeneration and glaucoma, highlights the clinical potential of targeting the VHL/HIF pathway. As such, the data presented here indicate that targeting HIFs for reprogramming is a promising therapeutic strategy for neurodegenerative diseases. Our findings demonstrate that suppressing glycolysis and enhancing mitochondrial flux in the RPE via genetic perturbations to the rods provided noncell-autonomous therapeutic benefits to photoreceptors. While we were unable to achieve metabolic reprogramming of the RPE, our work also supports the rationale for future endeavors aimed at suppressing glycolysis in the RPE via targeting of HIF1A. This insight supports the further investigation of glycolytic metabolism as a promising therapeutic approach. Such a strategy not only aims to address economic challenges associated with treating orphan disorders but also broadens the therapeutic scope for addressing the metabolic dysregulation at the heart of various neurodegenerative conditions, especially for patients with no identifiable genetic cause.

## Methods

### Sex as a biological variable

Sex was not considered as a biological variable. For all mouse studies conducted, both female and male mice were used.

### Preclinical models

*Pde6b^H620Q/H620Q^*; *Pde6g*^CreERT2/+^ is a previously reported preclinical model of retinitis pigmentosa (21, 52–54). *Pde6g*^Cre–ERT2^ mice were generated at Jonas Children’s Vision Care Laboratory (20, 52). *Vhl^loxP/loxP^* mice were obtained from Jackson Laboratory (strain no. 012933). *Pde6b^H620Q/H620Q^* and *Pde6g^CreERT2^* lines were crossed to yield experimental lines in this research study. *Pde6b^H620Q/H620Q^*; *Rpe65*^CreERT2/+^ mice were generated at Jonas Children’s Vision Care Laboratory, as previously reported (39). *Hif1a^loxP^^/loxP^* and *Hif2a^loxP^^/loxP^* mice obtained from Jackson Laboratory (strain no. 007561 and 008407, respectively). All mice were housed in the Edward S. Harkness Eye Institute at Columbia University Irving Medical Center Facility under a 12-hour light and 12-hour dark cycle (21, 55). Mice were euthanized following the Columbia University IACUC guidelines reported in previous studies (21, 55). Mice were used in accordance with the Statement for the Use of Animals in Ophthalmic and Vision Research of the Association for Research in Vision and Ophthalmology and the Policy on the Use of Animals in Neuroscience Research of the Society for Neuroscience.

### Tamoxifen injection

Per previous studies, tamoxifen (Sigma-Aldrich; T5648) was intraperitoneally injected 3 times between 7 and 10 days postnatal at a concentration of 100 μg/g body weight in the treatment group (20, 21). For the control group, a sham injection was administered with 10% Ethanol (w/w) in sunflower oil (Sigma-Aldrich; S5007) using methods described in Koch et al. and Zhang, et al. (21, 52–54).

### DNA recombination assay

To screen for genetic recombination and adequate excision, DNA was extracted from the whole retina via DNeasy Blood & Tissue Kit (Qiagen, 69506) at 3 weeks of age. PCR was performed as previously described (56). Primers that target *Vhl, Hif1a,* and *Hif2a* genomic regions are listed in Supplemental Table 1. The protein was extracted from the whole retina to assay for successful recombination. Immunoblots were performed as previously described to compare the protein levels of VHL, HIF1A, and HIF2A (56).

### Electroretinography

Mice were administered ERG tests using previously described methods (21, 30, 57). ERG testing was performed 4-, 6-, and 8-weeks of age. Mice were dark-adapted for at least 12 hours prior to assaying and anesthetized via IP injection with 0.1 mL/10 g body weight of 1 mL of 100 mg/mL ketamine and 0.1 mL of 20 mg/mL xylazine in 8.9 mL PBS (21). Mice were placed on heating pads to maintain their body temperature at 37°C throughout the study. Eyes were dilated with Tropicamide Ophthalmic Solution (1%; Akorn). Electrodes were placed on the corneas, which were subsequently protected by application of Gonak Hypromellose Ophthalmic Demulcent Solution (2.5%; Akorn). ERG recordings were measured simultaneously for both eyes. As previously described, pulses of 0.00130 cd/m2 and 3 cd/m2 (White-6500K) were employed (21, 52–54, 58). The described protocol allows for analysis of rod, cone, and mixed responses based on the length and intensity of light stimuli in addition to dark/light adaptation.

### Histology

Retinal morphology was studied via euthanasia and extraction of retinae from experimental or control mice stained with H&E as previously described at 4, 6, and 8 weeks of age (21, 52–54). Retinal ONL thickness was quantified from slide scanning of histological slides as a function of distance from the optic nerve head. By using Leica slide viewing software and included tools to measure the thickness of the outer nuclear layer, we were able to create “spidergrams” that map the thickness of the ONL as a distance from the optic nerve head. Spidergrams facilitated the comparison of thickness between treated and untreated regions within the same eye, as well as with control eyes that received no treatment.

### RNA-seq and analysis

Retinal and RPE/choroid tissues were collected and sent for RNA-seq at the New York Genome Center at 3 weeks of age. Sequencing, gene mapping, and count matrices were generated by the NYGC team. Results were analyzed using DESeq2 processing pipeline. Lowly expressed genes (those under 10 counts) were excluded from analysis. Volcano plots of DEGs were then generated using the EnhancedVolcano package. Heatmaps were generated via the Pheatmap package. Gene Ontology (GO) analysis to determine biological processes associated with DEGs was performed using the ClusterProfiler package.

### RNA Extraction and qRT-PCR

Total RNA was extracted from retinae using the RNeasy mini kit (QIAGEN, #74104) at 3 weeks of age and subsequently reverse transcribed to generated cDNA using SuperScript III First-Strand Synthesis SuperMix (Thermo Fisher Scientific, 18080-400). The reactions were run as previously described (56). Transcript levels of each target gene were determined by SYBR Green-based qPCR (BIO-RAD, 1725271) and were standardized to *β-Actin*. Primers are shown in Supplemental Table 2.

### Immunoblotting of retinal lysates

Retinas were harvested and prepared for immunoblot at 3 weeks of age prior to disease onset using previously described experimental procedures (21). The protein lysate was resuspended, and the supernatant was collected and subjected to SDS-PAGE using 4%–15% BIO-RAD TGX precast gels (4561083), followed by transferring to nitrocellulose membranes for immunoblotting analysis. Membranes were blocked in blocking buffer at room temperature for 30 minutes and incubated with primary antibodies overnight at 4**°**C, washed, and subsequently incubated with the appropriate reactive secondary antibody, dependent on the primary antibody’s host species, at room temperature for 1 hour. Immunoblotting signals were visualized by an iBright FL 1500 Imaging System (Thermo Fisher Scientific). Whole-cell proteins were normalized to β-Actin (Cell Signaling, 3700). A complete list of antibodies is found in Supplemental Table 3.

### Metabolomics and isotopic tracing

#### Metabolic flux.

To measure glycolytic and oxidative phosphorylation intermediates following the loss of VHL, we have quantified metabolic flux levels in treated (*Vhl*^–/–^; *Pde6g^CreERT2/+^*; *Pde6b^H620Q/H620Q^*) and control *(Vhl^loxP/loxP^; Pde6g^CreERT2/+^; Pde6b^H620Q/H620Q^)* retinae using stable isotope–resolved metabolomics coupled with gas chromatography/mass spectrometry (GC-MS) at 3 weeks of age, as previously reported (20, 21, 58). Both in vivo and ex vivo samples were analyzed to evaluate how perturbed components following VHL ablation behaved independently and within the context of the highly intertwined retinal ecosystem. Targeted analysis was focused on glycolysis, mitochondrial activity, and other potentially relevant metabolic pathways.

#### In vivo analysis.

Treated and untreated mice were injected intraperitoneally with U-^13^C glucose (500 mg/Kg). The mice were then closely monitored and euthanized at the appropriate 5-, 15-, 30-, and 45- minute time points. The eyes were rapidly enucleated and immediately snap frozen on liquid nitrogen for downstream metabolic processing via GC-MS.

#### Ex vivo analysis.

Mice were euthanized, and retinae were extracted and quickly dissected for either the neuroretina or RPE/choroid. They were subsequently balanced in a Krebs-Ringer Bicarbonate buffer supplemented with 5 mM glucose for 15 minutes. Afterward, the retinae were exposed to U-^13^C glucose solutions for the time of interest, before being washed to remove residual solution and snap frozen on liquid nitrogen for analysis. Metabolites were extracted with 80% methanol, derivatized for GC-MS, and quantified as previously described (20, 21).

### Cone densities

Whole retinae were dissected and flat-mounted, as previously described (59–61) at approximately 10 weeks of age. Cone numbers were counted within each tile and subsequently averaged and compared (54). Regions throughout the retina were analyzed to determine if there was a spatially relevant context for rescue.

### Statistics

To determine statistical significance in our analyses, a standard 2-tailed *t* test with equal variance assumptions was deployed when explicitly comparing 2 groups. When comparing multiple experimental conditions (such as in Figure 3A), a 1-way ANOVA was initially performed to determine if a difference existed between conditions followed by appropriate post hoc Tukey tests to determine which groups differed. Bulk RNA-seq statistical analyses were performed within DESeq2, which deploys Wald tests and corrects for multiple comparisons with the Benjamini-Hochberg method to control the false discovery rate (FDR). Across the statistical methods described above, a *P* value (adjusted where appropriate) of less than 0.05 was considered significant. All ERG results are averaged across the 2 studied eyes, with each mouse representing *n* = 1. RNA and Protein analysis considers a single retinal extract as *n* = 1. All data analysis was performed in PRISM GraphPad.

### Study approval

All animal studies were conducted in accordance with and approved by the Institutional Animal Care and Use Committee Protocol Number AABU2668 at Columbia University Irving Medical Center.

### Data availability

All underlying data can be accessed within the Supporting Data Values file. All bulk RNA-seq data are deposited at the NCBI’s Gene Expression Omnibus database (GEO GSE287734). Additional information can be obtained from the corresponding authors upon request.

## Author contributions

The order of the co–first authors was determined based on their efforts and contributions to the study. SMC, XC, and SHT conceptualized the study. SMC, XC, BMR, NH, AD, BLDC, DTH, PMJQ, JD, JBH, and SHT developed the methodology. SMC, XC, and NH performed the investigation. SMC and XC were responsible for visualization. SHT and JBH acquired funding. SHT was the project administrator. SMC, XC, JBH, and SHT supervised the project. SMC and SHT wrote the original draft. SMC, BLDC, DTH, PMJQ, JBH, and SHT reviewed and edited the manuscript.

## Supplementary Material

Supplemental data

Unedited blot and gel images

Supporting data values

## Version 1 02/12/2025

In-Press Preview

## Version 2 04/01/2025

Electronic publication
