# Supplementary material for: Ablating VHL in rod photoreceptors modulates RPE glycolysis and improves preclinical model of retinitis pigmentosa
Source: J Clin Invest. 2025 Feb 12;135(7):e185796. doi: 10.1172/JCI185796 (PMC11957697; doi:10.1172/JCI185796)
Supplement: Supplemental data [file jci-135-185796-s125.pdf]

Supplemental Tables:

Supplemental Table 1: Genomic PCR primers used for amplification and validation of VHL and HIF Loci.

Primer Application	Primer Sequence
Vhl Common Reverse Primer	CTGACTTCCACTGATGCTTGTCACAG
Vhl Single LoxP Forward Primer	CTGGTACCCACGAAACTGTC
Vhl Dual LoxP Forward Primer	CTAGGCACCGAGCTTAGAGGTTTGCG
Hif1a Forward Primer	GGTGCTGGTGTCCAAAATGT
Hif1a Reverse Primer	GGGCAGTACTGGAAAGATGG
Hif2a Common Reverse Primer	CAGGCAGTATGCCTGGCTAATTCCAGTT
Hif2a Single LoxP Forward Primer	CTTCTTCCATCATCTGGGATCTGGGACT
Hif2a Dual LoxP Forward Primer	GCTAACACTGTACTGTCTGAAAGAGTAGC

Supplemental Table 2: qPCR primers used for amplification and assessment of relative transcriptional levels.

Primer Application	Primer Sequence
Vhl Primer Forward	TCCACAGCTACCGAGGTCAT
Vhl Primer Reverse	TTCCGCACACTTGGGTAGTC
Hif1a Primer Forward	CTTGACAAGCTAGCCGGAGG
Hif1a Primer Reverse	CGACGTTCAGAACTCATCCTATTTT
Hif2a Primer Forward	GGTCATCGCAGTTGGAACCT
Hif2a Primer Reverse	GAAGTCCTTTGCAGACCTCATC
Glut1 Primer Forward	CACTGTGGTGTCGCTGTTTG
Glut1 Primer Reverse	AAAGATGGCCACGATGCTCA
Glut2 Primer Forward	ACCGGGATGATTGGCATGTT
Glut2 Primer Reverse	CCCAAGGAAGTCCGCAATGT
Glut3 Primer Forward	CCTCAGCTGCAGCCTACTT
Glut3 Primer Reverse	ATGTCCTCGAAAGTCCTGCC
Glut4 Primer Forward	CCATCTTGATGACCGTGGCT
Glut4 Primer Reverse	ACCCATAGCATCCGCAACAT
Hk1 Primer Forward	AAGGAGACCAACAGCAGAGC
Hk1 Primer Reverse	AAGTCACCATGCTCAGTCCC
Pfkm Primer Forward	TCGCGATCTCCAGGTGAATG
Pfkm Primer Reverse	CTGTCAAAGGGAGTTGGGCT
Pfkp Primer Forward	GGGGCCTCGTACTCAGAAAC
Pfkp Primer Reverse	CCCTTCAGTTTGGCCGAGAT
Pfkl Primer Forward	GGTGTTTGCCAATGCTCCAG
Pfkl Primer Reverse	GGCATGCGGTGCTCAAAATC
Pkm1 Primer Forward	TCGCATGCAGCACCTGATAG

Pkm1 Primer Reverse	AGGTCTGTGGAGTGACTGGA
Pkm2 Primer Forward	CATGCAGCACCTGATTGCCC
Pkm2 Primer Reverse	CCACTGCAGCACTTGAAGGA
Ldha Primer Forward	CGTGCACTAGCGGTCTCAAA
Ldha Primer Reverse	CTTGTTCTGGGGAGCCTGC
Ldhb Primer Forward	CTCCTCCTTCTTGTAGAGCCG
Ldhb Primer Reverse	GGGTTGCCATCTTGTCCAGAA
β-Actin Primer Forward	CACTGTCGAGTCGCGTCC
β-Actin Primer Reverse	TCATCCATGGCGAACTGGTG

Supplemental Table 3: Primary and Secondary Antibodies Used for Immunoblotting.

Antibody Information	Dilution Factor
Von Hippel Lindau/VHL Antibody (Santa Cruz Biotechnology, sc-17780)	1:250
HIF1A Antibody (Novus Biologicals, NB100-105)	1:500
HIF2A/EPAS1 Antibody (Novus Biologicals, NB100-122)	1:1000
Anti-Glucose Transporter GLUT1 antibody (Abcam, ab40084)	1:1000
Anti-Glucose Transporter GLUT2 antibody (Abcam, ab54460)	1:1000
Muscle Phosphofructokinase/PFKM/PFK-1 Antibody (Novus Biologicals, NBP1-87293)	1:500
PDK1 Antibody (Cell Signaling Technologies, 3062S)	1:1000
LDHA Antibody (Cell Signaling Technologies, 2012S)	1:1000
SLC16A3/MCT4 Polyclonal Antibody (Invitrogen, PA5-106683)	1:1000
β-Actin (8H10D10) Mouse mAb (Cell Signaling Technology, 12262S)	1:1000
Donkey anti-Mouse IgG (H+L) Secondary Antibody, HRP (Invitrogen, A16011)	1:5000
Donkey anti-Rabbit IgG (H+L) Cross-Adsorbed Secondary Antibody, HRP (Invitrogen, 31458)	1:5000

Supplemental Figures:

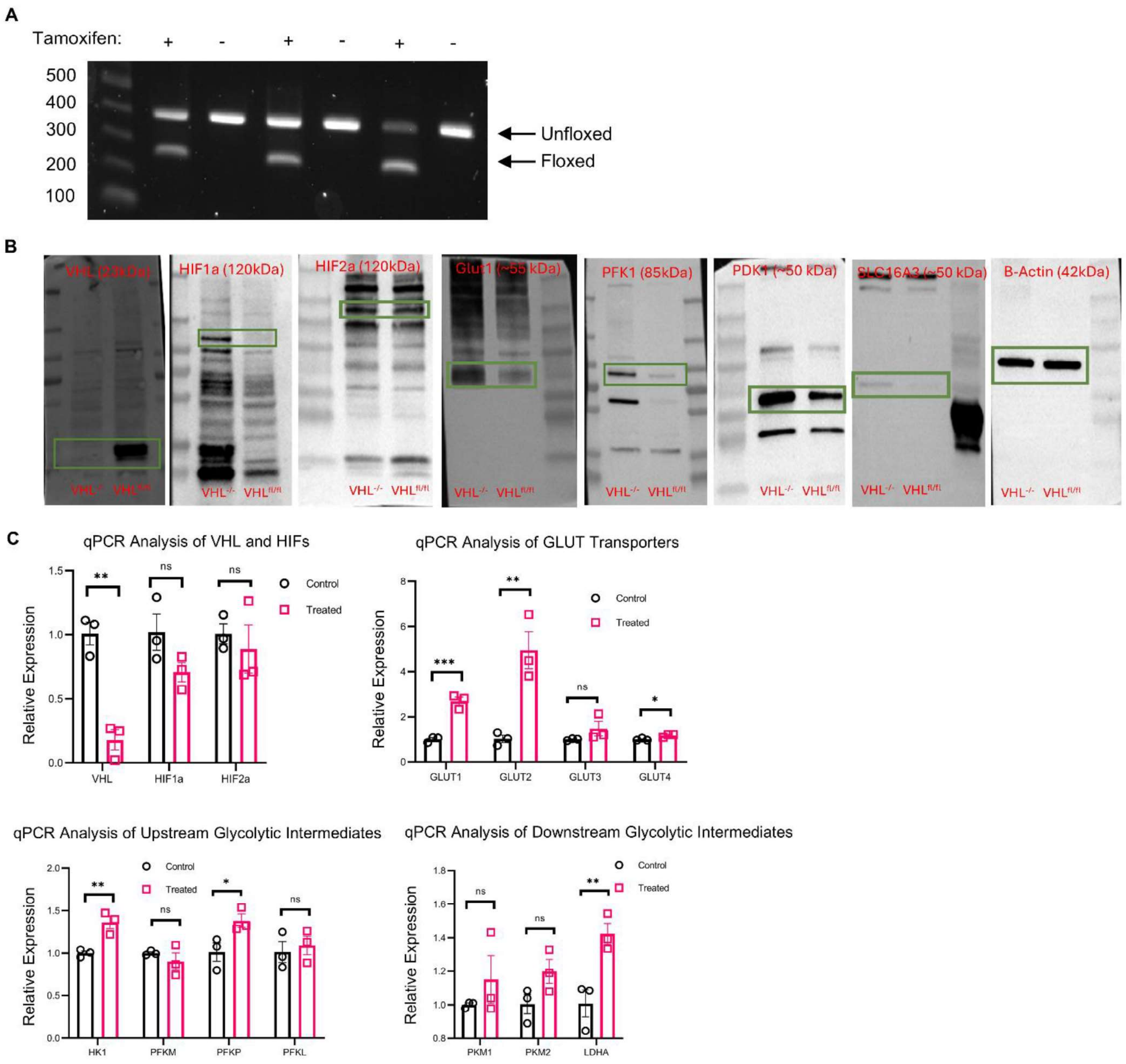

Supplemental Figure 1. Molecular validation of VHL ablation in photoreceptors reveals alterations at the genomic and proteomic levels. Molecular validation of VHL ablation supports previous findings regarding the roles of HIFs and their downstream targets while demonstrating the fidelity of our Cre-loxP system. (**A**) PCR amplification of tamoxifen injected (treated) and uninjected (control) mice at the VHL genomic loci demonstrated clear truncation only in the treated population. (**B**) Immunoblots of VHL, HIFs, and their well-established downstream targets such as phosphofructokinases and glucose transporters supported the proposed mechanism of rescue. Mice were evaluated at 3 weeks of age prior to disease onset. (**C**) qPCR validation of RNA-seq findings demonstrated consistent upregulation of glucose transporters (*Glut1*), upstream glycolytic enzymes (*Hk1*, *Pkfp*), and downstream enzymes (*Ldha*). All results were normalized with respect to *β-Actin* levels and analyzed using double delta Ct methods (N=3). Mice were evaluated at 3 weeks of age and confirmed proteomic perturbations identified in immunoblots.

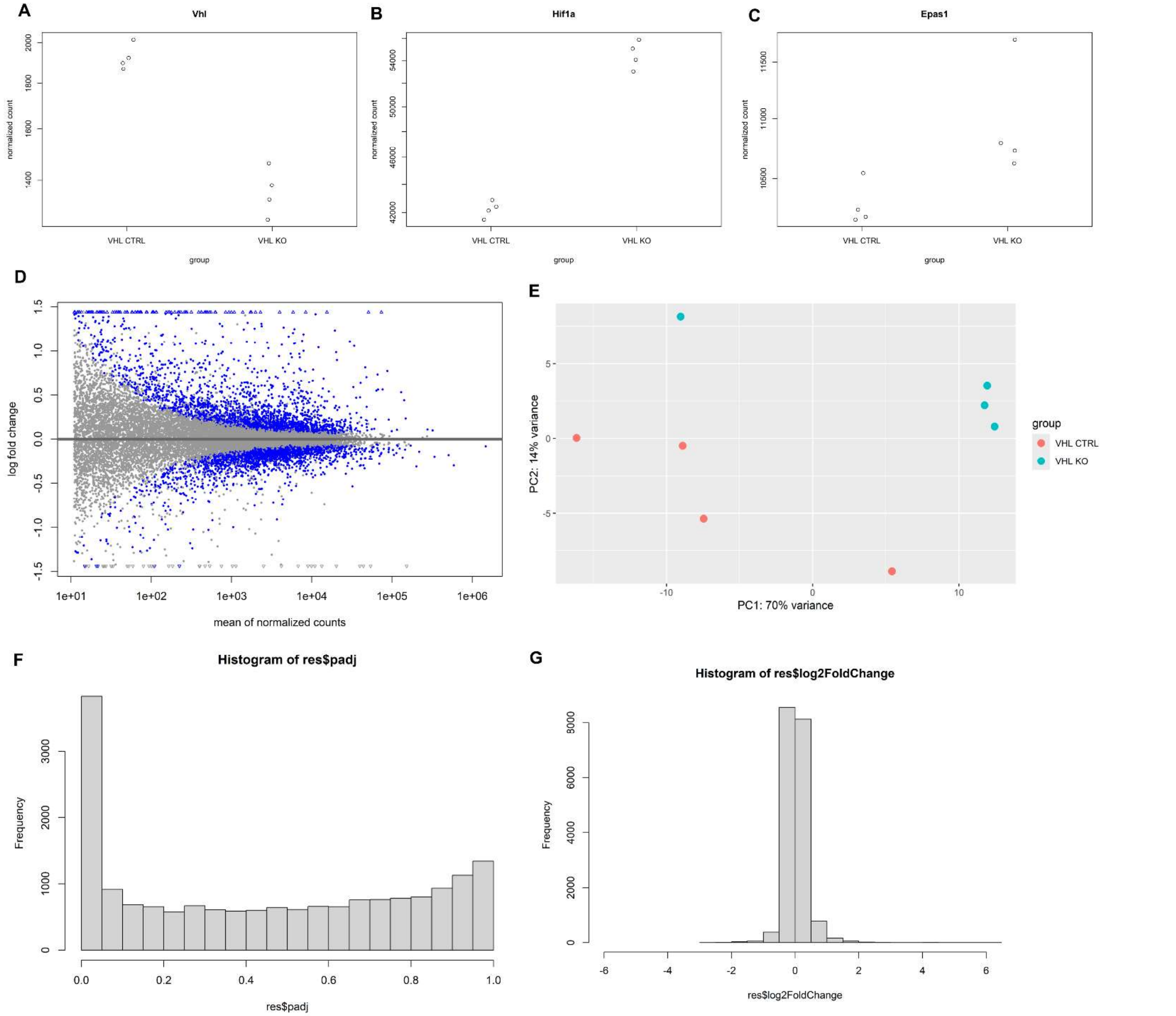

Supplemental Figure 2. Supporting analyses of bulk RNA-seq comparing VHL ablated and unablated neuroretinas. Supporting graphs and data analysis justifying bulk RNA-seq findings including target engagement of VHL (**A**), HIF1A (**B**), and HIF2A (**C**). (**D**) MA plot, principal component analysis (**E**), and histograms of adjusted P value (**F**) and Log_2(Fold Change) (**G**) are also provided.

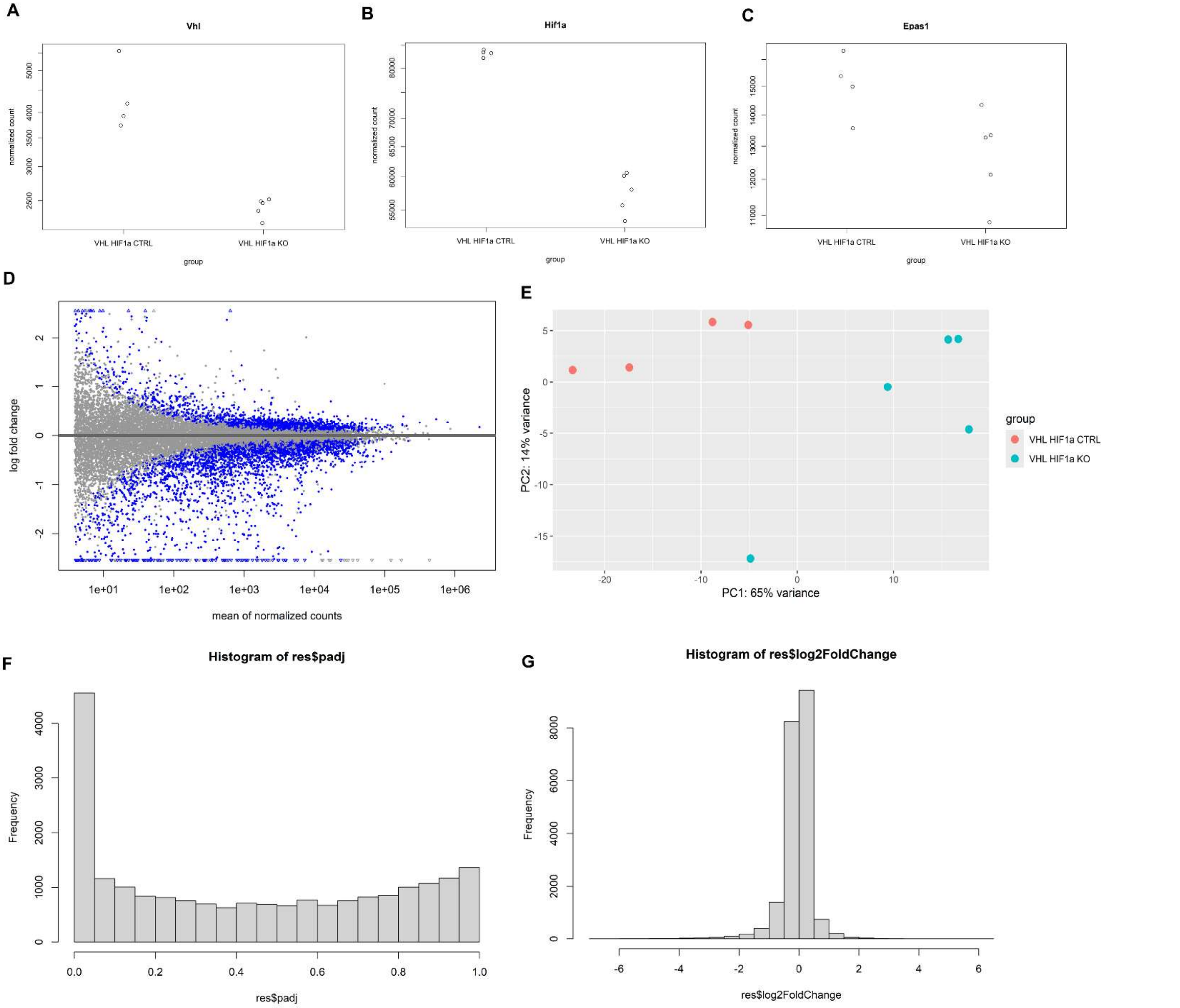

Supplemental Figure 3. Supporting analyses of bulk RNA-seq comparing control and VHL/HIF1A ablated neuroretinas. Supporting graphs and data analysis justifying bulk RNA-seq findings including target engagement of VHL (**A**), HIF1A (**B**), and HIF2A (**C**). (**D**) MA plot, principal component analysis (**E**), and histograms of adjusted P value (**F**) and Log_2(Fold Change) (**G**) are also provided.

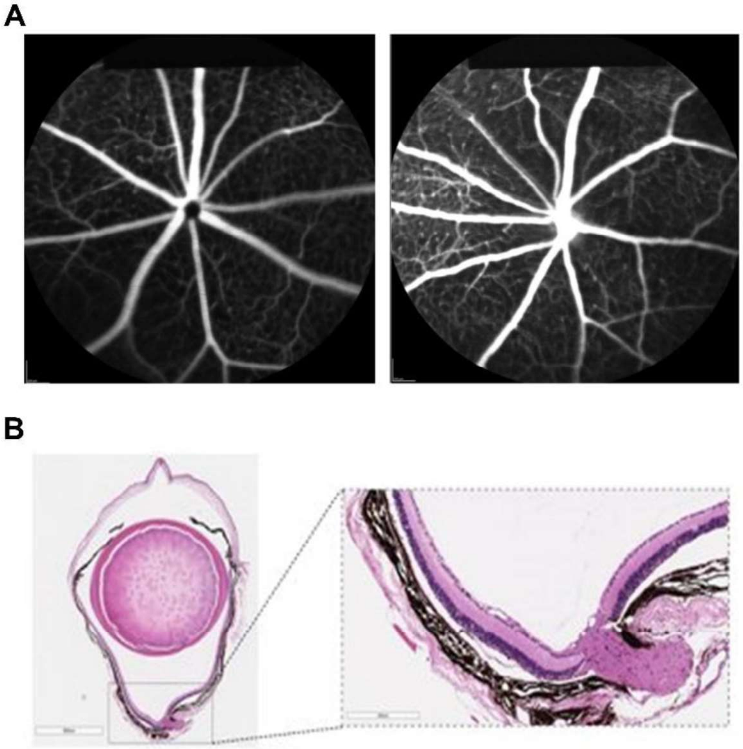

Supplemental Figure 4. VHL ablation in rod photoreceptors does not result in abnormal vascularization or blastoma formation. Given the connection between HIFs and angiogenic growth factors, we explored the potential for abnormal vascularization and hemangioblastoma formation, observing no significant findings or adverse events. (**A**) Angiograms of treated (left) and untreated (right) mice at three weeks of age prior to disease onset where no significant findings were identified. (**B**) Histological images of treated mice at 18 months of age used to determine the presence of hemangioblastomas or other malignant formations. No adverse events were detected.

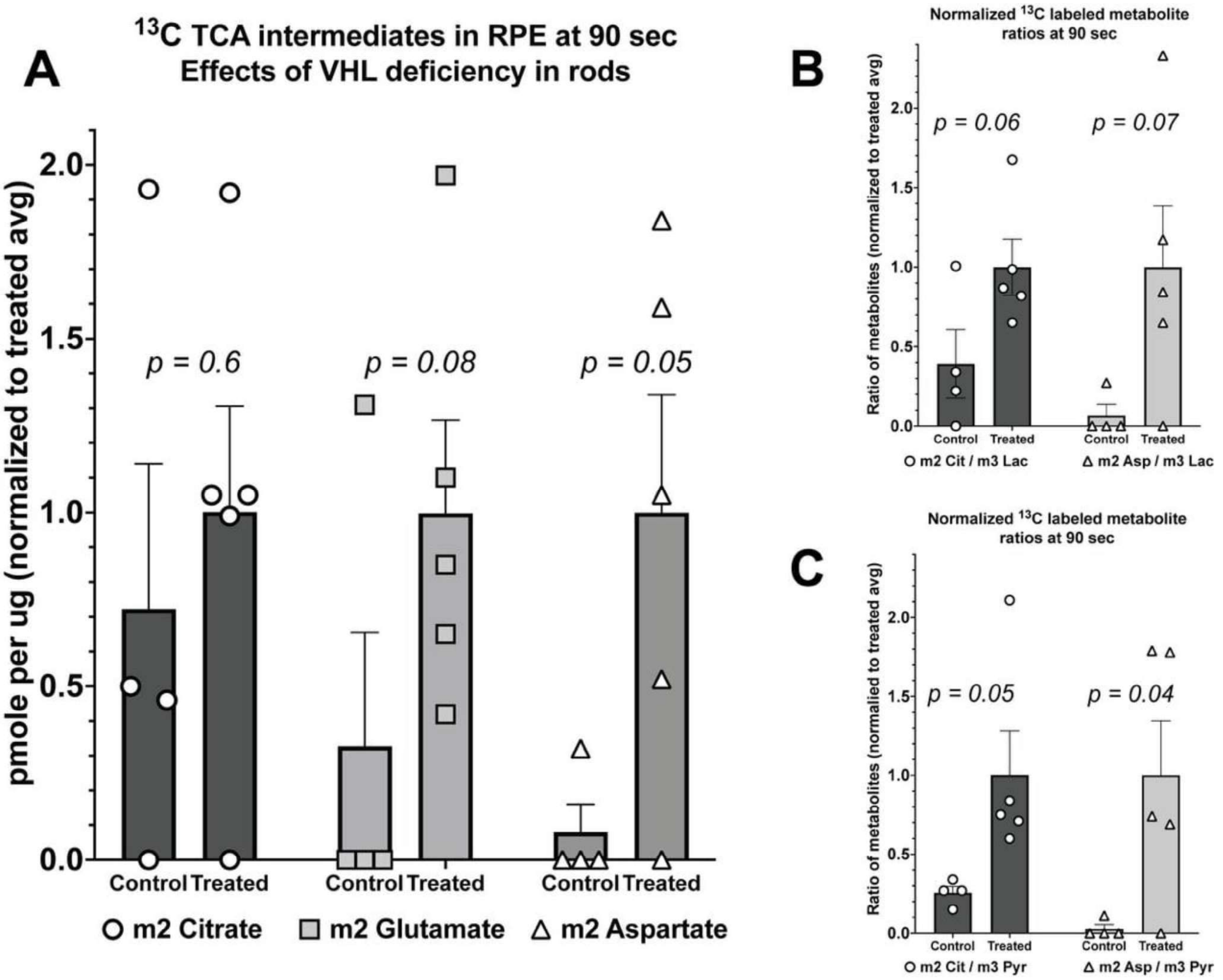

Supplemental Figure 5. VHL ablation in rods resulted in non-cell-autonomous increase in mitochondrial TCA in genetically unperturbed RPE. A more detailed statistical analysis of the 90 second data of the experiments shown in Figure 4C confirmed that the loss of VHL specifically only in rods causes a secondary effect on RPE choroid tissue that persists when the RPC/choroid tissue is isolated from the eye. (**A**) Metabolic flux from U-^{13}C glucose through glycolysis and the TCA cycle in REP/choroid isolated from VHL ablated and unablated rod photoreceptor cells. (**B**) and (**C**) Ratios of ^{13}C citrate and ^{13}C aspartate to either ^{13}C lactate or ^{13}C pyruvate at 90 seconds (N>=4).

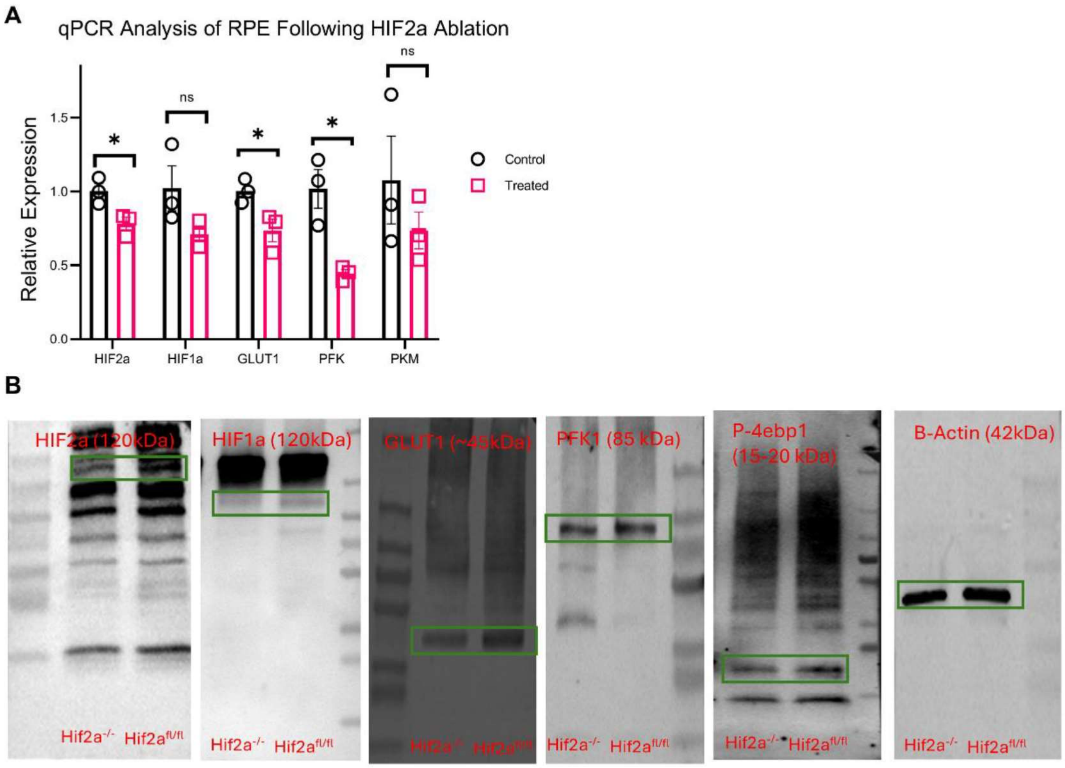

Supplemental Figure 6. Molecular validation of HIF2A ablation in RPE demonstrates target engagement and fidelity of treatment. qPCR and immunoblotting of known HIF2A targets in 3-week-old retinas demonstrate changes at the transcriptional and proteomic levels indicative of successful target engagement following tamoxifen injection. (**A**) qPCR of HIF2A and known downstream HIF2A targets such as GLUT1 demonstrate a transcriptional perturbation in response to cell-specific KO that is in-line with previously explored relationships (N=3). (**B**) Immunoblots of the RPE for HIFs demonstrated downregulation of HIF2A and known targets.

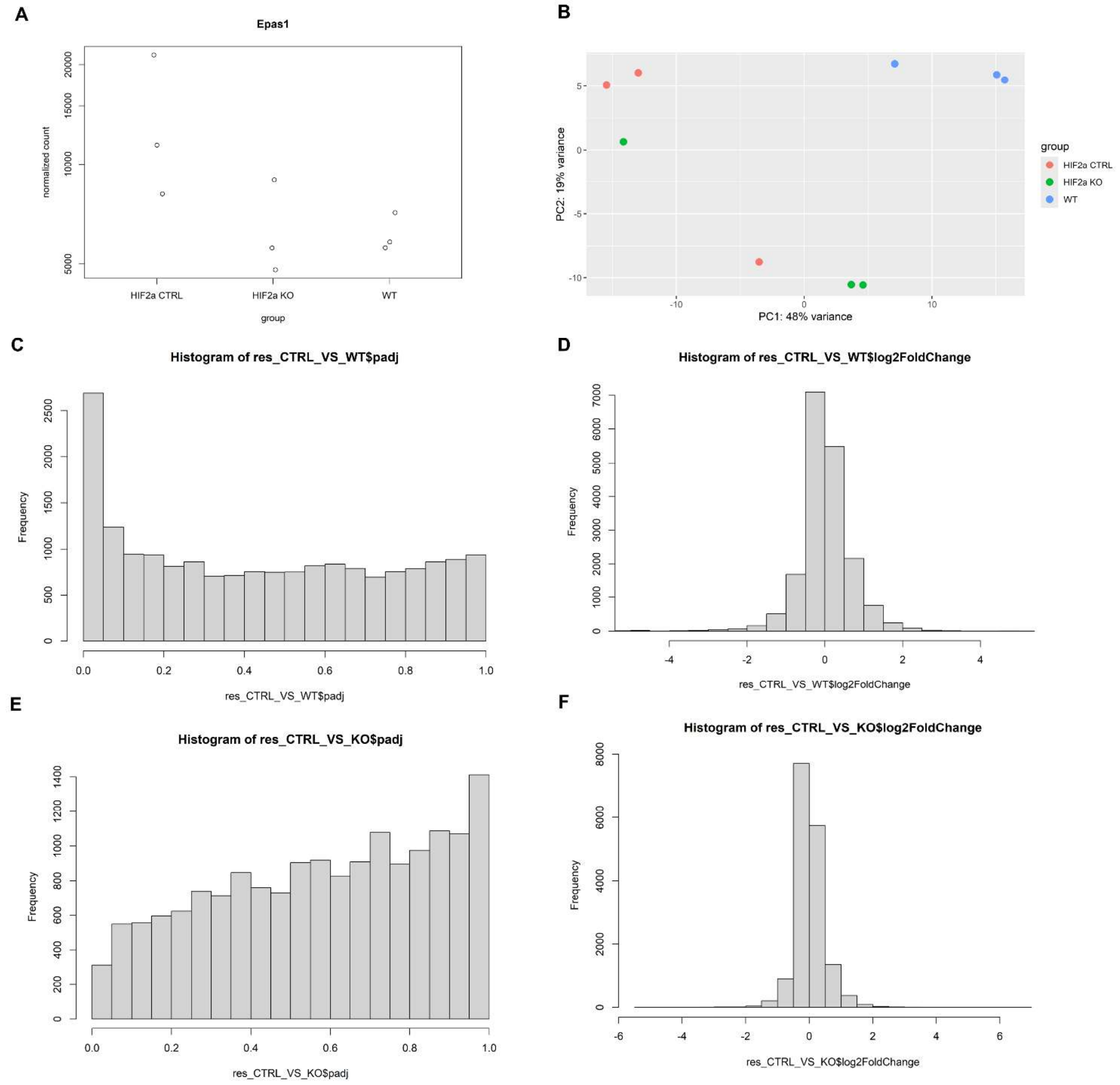

Supplemental Figure 7. Supporting analyses of bulk RNA-seq comparing RPE/Choroid of wildtype, diseased, and diseased mice with HIF2A ablated in the RPE. Supporting graphs and data analysis justifying bulk RNA-seq findings including target engagement of VHL HIF2A (**A**). (**B**) principal component analysis and histograms of adjusted P value (**C**) and Log_2(Fold Change) (**D**) for wildtype vs diseased mice in addition to histograms of adjusted P value (**E**) and Log_2(Fold Change) (**F**) for diseased vs diseased with HIF2A KO in RPE are shown.

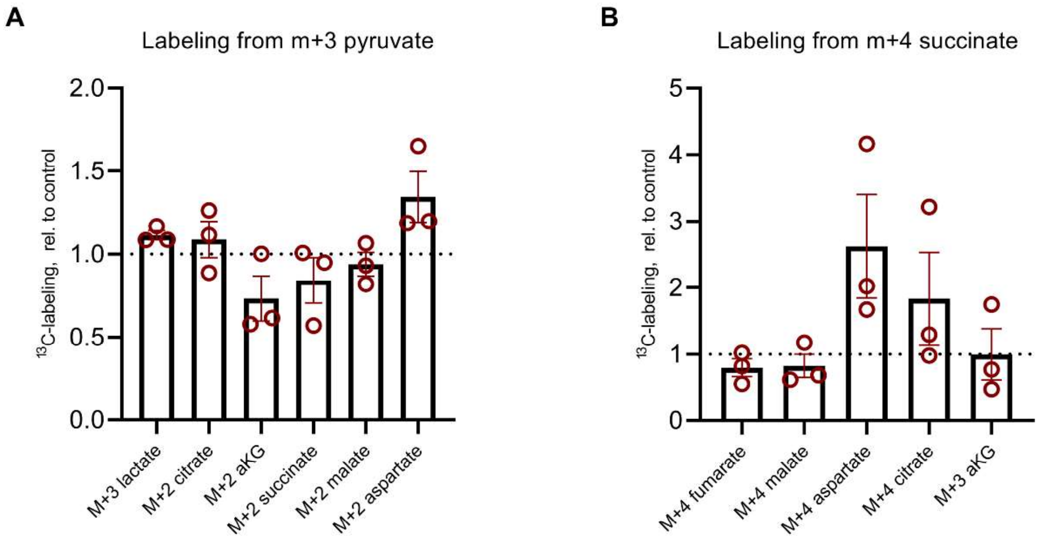

Supplemental Figure 8. ^{13}C succinate labeling showed enhanced mitochondrial flux in the RPE. *Ex vivo* analysis of HIF2A ablated and unablated RPE demonstrated minor differences. (**A**) Metabolic tracings from RPE/choroid tissues dipped in 5mM U-^{13}C pyruvate for 90 seconds indicated no overall change in flux. (**B**) Metabolic tracings from RPE/choroid tissues dipped in 5mM U-^{13}C succinate for 90 seconds identified no statistically significant difference between control and experimental groups (n=3), but trends positively towards an increase in mitochondrial activity as shown by elevated M+4 aspartate following HIF2A KO. Error bars represent S.E.M.

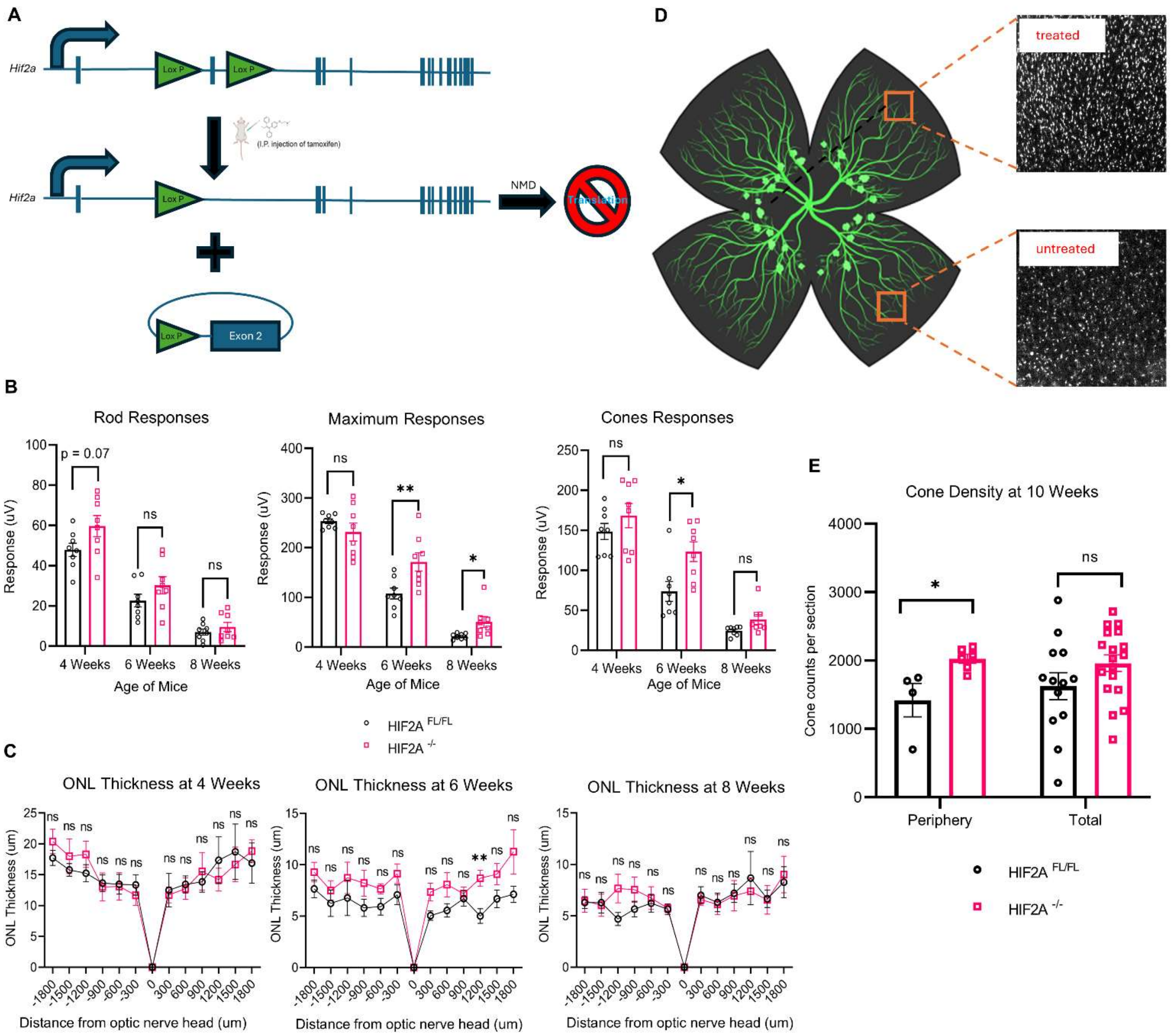

Supplemental Figure 9. RPE-specific ablation of HIF2A offers therapeutic benefits and increases cone lifespan. RPE-specific ablation of HIF2A results in transient functional benefits and preserves cone cells over an extended period. **(A)** General diagram of genetic recombination that occurs in mice floxed for exon 2 of *Hif2a* following tamoxifen injection. (**B**) ERG analysis of control (*Hif2a*$^{loxP/loxP}$; *Pde6b*$^{H620Q/H620Q}$; *Rpe65*$^{P2A\text{-}Cre\text{-}ERT2/+}$) and treated (*Hif2a*$^{-/-}$; *Pde6b*$^{H620Q/H620Q}$; *Rpe65*$^{P2A\text{-}Cre\text{-}ERT2/+}$) mice injected with tamoxifen (N=8). (**C**) Spidergrams depicting thickness of ONL as a function of distance from the optic nerve heard estimated histological preservation and treatment effect (N>=5). (**D**) Graphic depicting peanut agglutinin (PNA) staining of cones in 10-week-old control and treated mice. **(E)** Bar graph depicting the counts of cone cells measured in the periphery and averaged over the entirety of the retina (N>=4). Error bars represent S.E.M. * $p < 0.05$; ** $p < 0.01$.
