# Supplementary figures and images for: Ablating VHL in rod photoreceptors modulates RPE glycolysis and improves preclinical model of retinitis pigmentosa
Source: J Clin Invest. 2025 Feb 12;135(7):e185796. doi: 10.1172/JCI185796 (PMC11957697; doi:10.1172/JCI185796)

Uncropped Blots for Supplemental Figure 1B

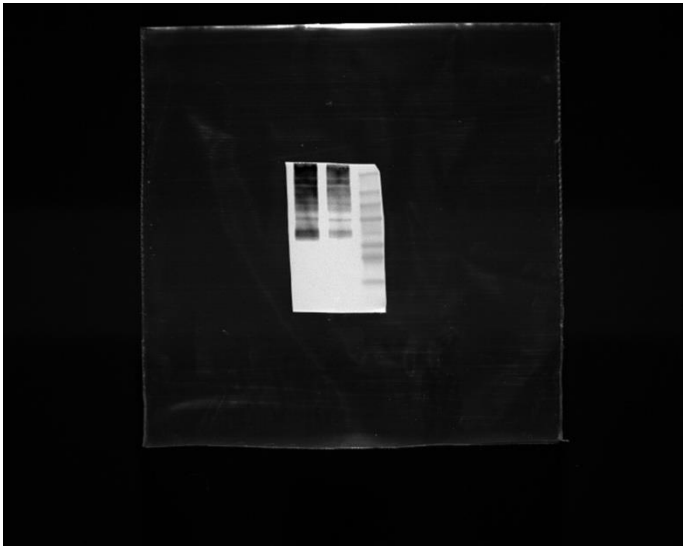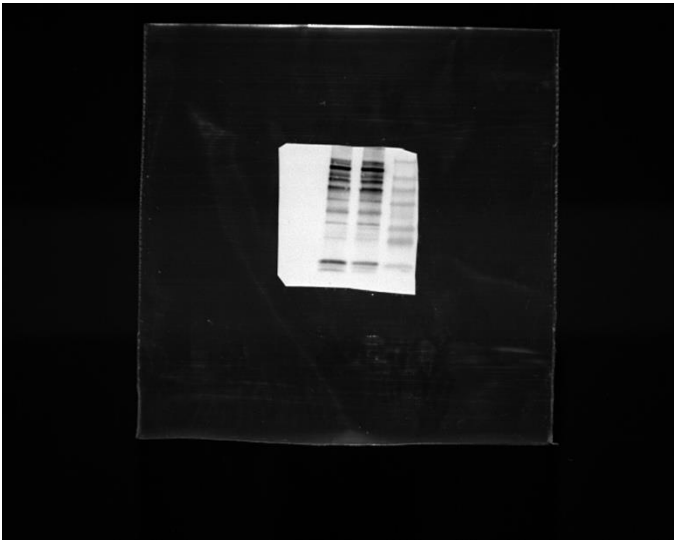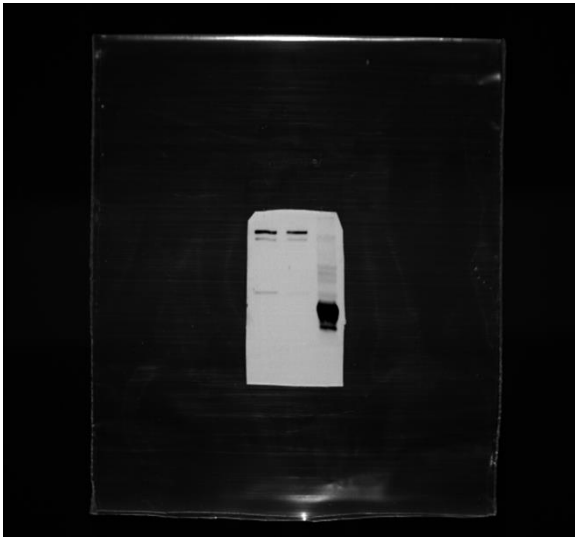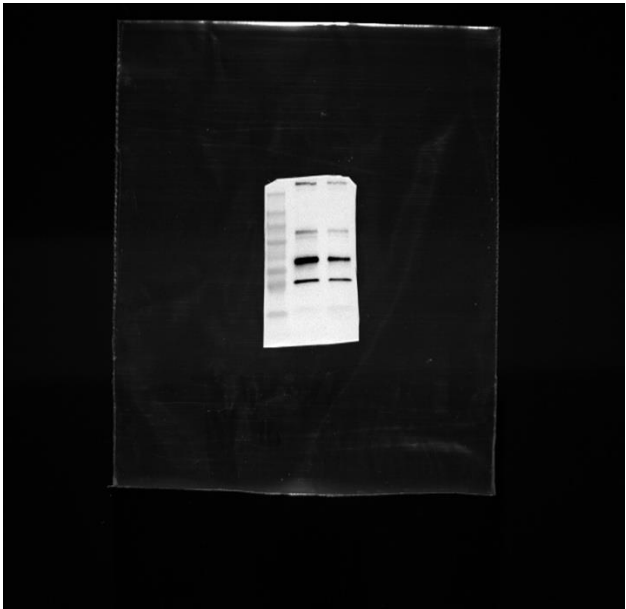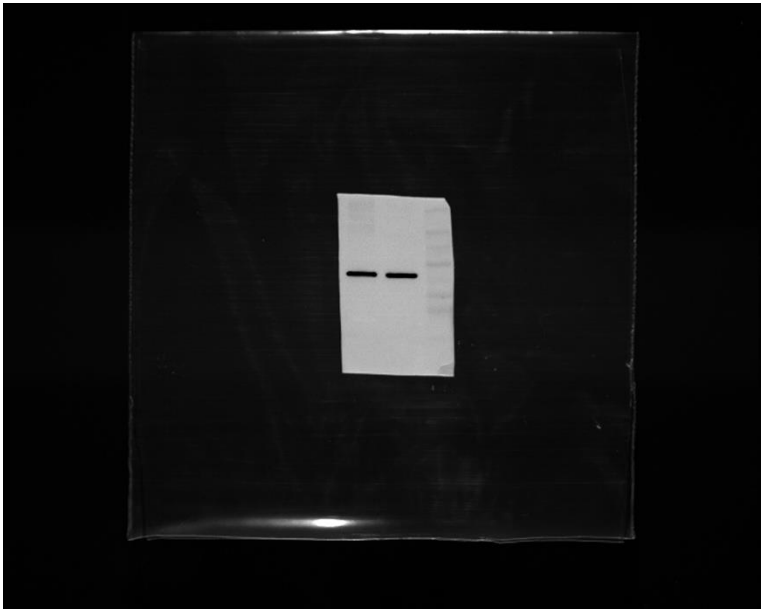

Uncropped Blots for Supplemental Figure 6B

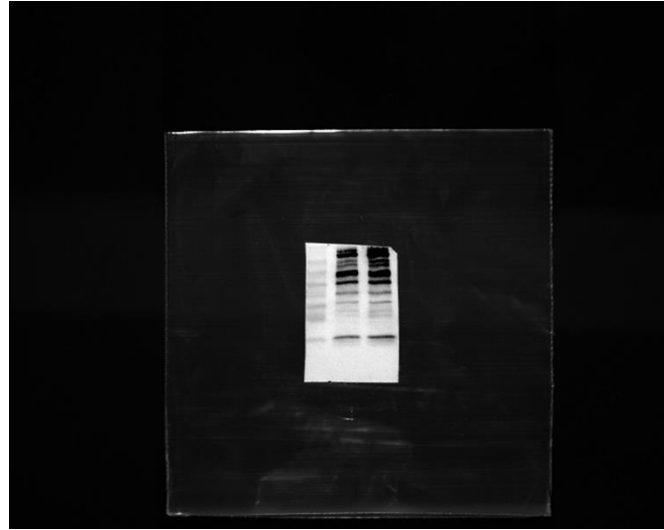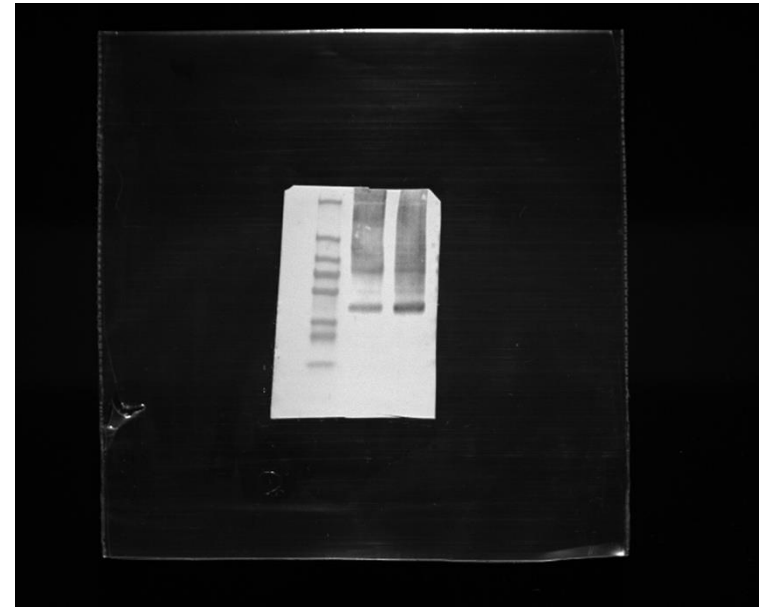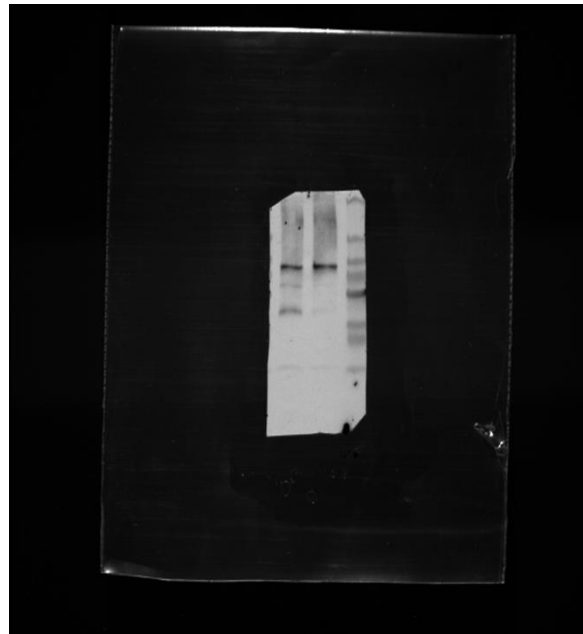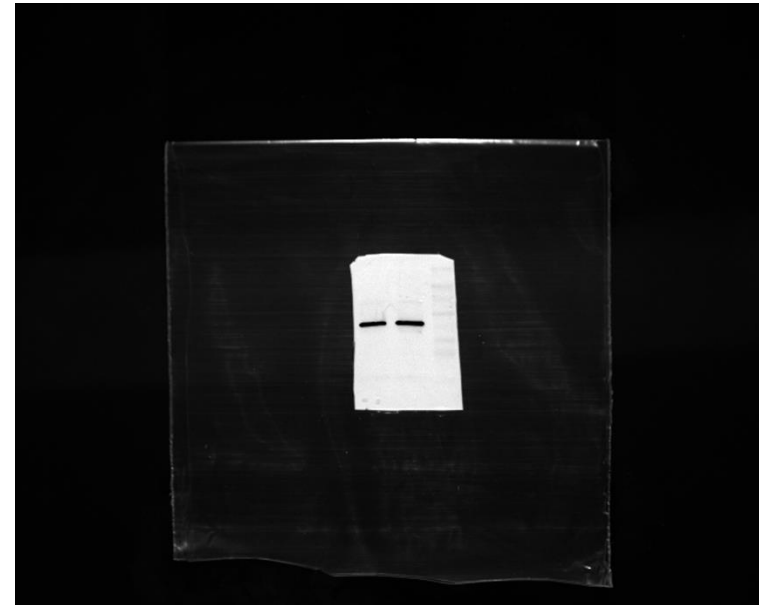

Supplement: Unedited blot and gel images [file jci-135-185796-s126.pdf]
